# Recent advances in nanomaterial-driven strategies for diagnosis and therapy of vascular anomalies

**DOI:** 10.1186/s12951-024-02370-2

**Published:** 2024-03-18

**Authors:** Yiming Geng, Huwei Zou, Zhaowei Li, Haiwei Wu

**Affiliations:** 1 Department of Oral and Maxillofacial Surgery, Shandong Provincial Hospital Affiliated to Shandong First Medical University, 324 Jingwu Road, Jinan, 250021 China; 2 School of Radiology, Shandong First Medical University and Shandong Academy of Medical Sciences, 619 Changcheng Road, Tai’an, 271000 China

**Keywords:** Vascular tumors, Vascular malformations, Contrast agents, Nanomedicine, Biomaterial

## Abstract

Nanotechnology has demonstrated immense potential in various fields, especially in biomedical field. Among these domains, the development of nanotechnology for diagnosing and treating vascular anomalies has garnered significant attention. Vascular anomalies refer to structural and functional anomalies within the vascular system, which can result in conditions such as vascular malformations and tumors. These anomalies can significantly impact the quality of life of patients and pose significant health concerns. Nanoscale contrast agents have been developed for targeted imaging of blood vessels, enabling more precise identification and characterization of vascular anomalies. These contrast agents can be designed to bind specifically to abnormal blood vessels, providing healthcare professionals with a clearer view of the affected areas. More importantly, nanotechnology also offers promising solutions for targeted therapeutic interventions. Nanoparticles can be engineered to deliver drugs directly to the site of vascular anomalies, maximizing therapeutic effects while minimizing side effects on healthy tissues. Meanwhile, by incorporating functional components into nanoparticles, such as photosensitizers, nanotechnology enables innovative treatment modalities such as photothermal therapy and photodynamic therapy. This review focuses on the applications and potential of nanotechnology in the imaging and therapy of vascular anomalies, as well as discusses the present challenges and future directions.

**Graphical Abstract:**

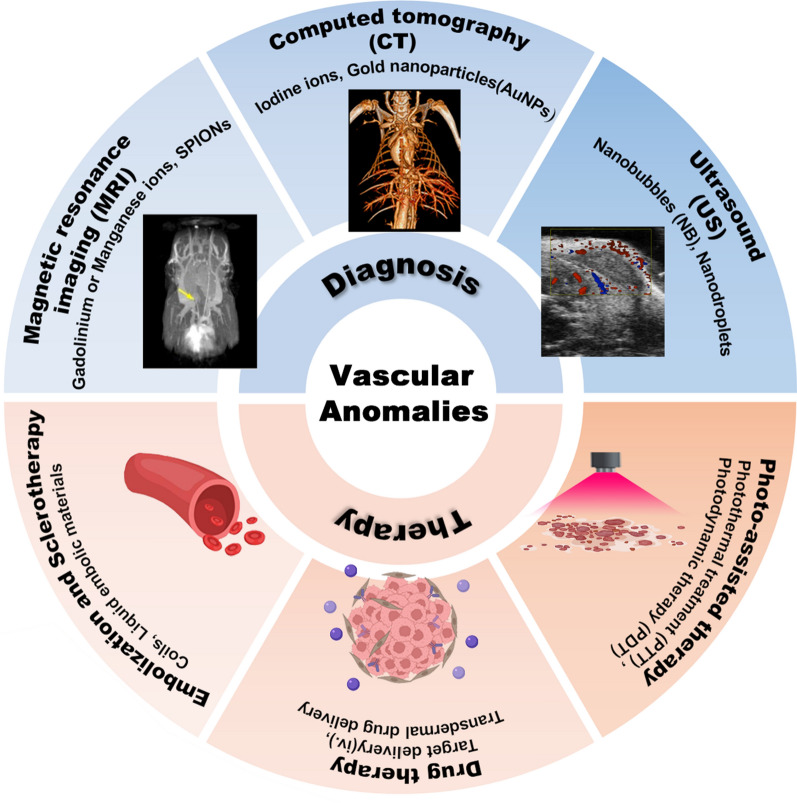

## Introduction

Vascular anomalies are recognized to be the most complicated vascular disease. Vascular anomalies include various diseases, which are mainly classified into two types according to the International Society for the Study of Vascular Anomalies (ISSVA): vascular tumors (e.g., hemangiomas) with proliferative changes of endothelial cells, and vascular malformations primarily consisting of structural vascular anomalies [1]. These encompassed a wide range from simple irregularities to complex structures involving arteries, veins, and lymphatics. Vascular anomalies can affect any part of the body, but lesions in the head and neck need particular attention because lesions have the potential to generate negative effects on both normal physiological and psychological health. Depending upon the location, vascular anomalies can always result in disfigurement and functional impairment. Vascular tumors result from proliferating endothelial cells possessing proliferative ability and malignant potential. Most vascular malformations exist at birth or shortly after birth and can gradually enlarge during lifetime of patients with novel lesions appearing. Due to the complex characteristics of vascular anomalies, clinical and radiologic diagnosis is essential for guiding appropriate treatment [2].

Considering the emerging complexity, multifactoriality, and challenges of vascular anomalies associated with vascular and lymphatic systems, numerous methods have been applied in therapy. Traditional treatment approaches for vascular anomalies mainly include surgical resection or radiation therapy, but they often come with limitations such as invasiveness and incomplete therapeutic outcomes [3]. In light of these challenges, nanotechnology offers a new frontier for diagnosing and treating vascular anomalies. A variety of targeted combination therapies and precision nanomedicine strategies have been developed, including those based on composite bioresponsive and biomimetic drug delivery nano-systems, for the more specialized, secure, and successful therapy of multifactorial disorders, avoiding off-targeted drug delivery and toxicity to healthy tissues, drug resistance phenomenon, uncontrolled release. In addition, nano-systems have the potential to modify traditional clinical materials, leading to excellent performance and structural alterations, thereby achieving the possibility of combined therapeutic effects [4]. Recent findings consistently showed that novel nanomedicine research and application methodologies could identify and treat complex vascular disorders both directly and indirectly.

**Scheme 1:**
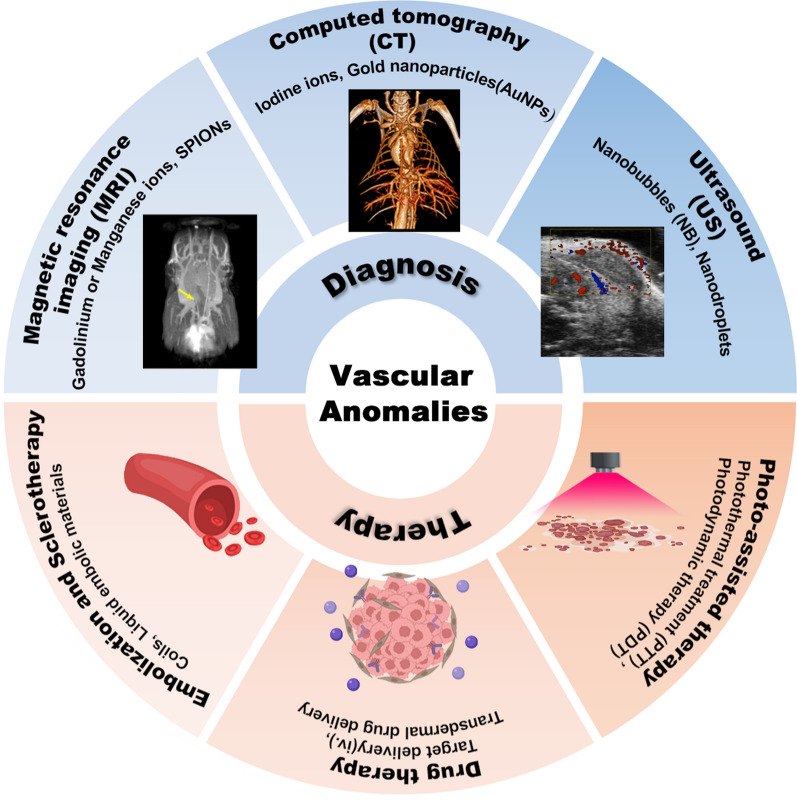
Advances in biotechnology and nanomaterial science have facilitated the development of the diagnosis and therapy of vascular anomalies

In this review, we first summarized the recent advances in the development of nano-systems or biomaterials with diagnostic, therapeutic, or combined properties, emphasizing their particle designs, therapeutic effects, as well as challenges and limitations. The nano-systems offer safer and more effective diagnosis, treatment, and outcome monitoring methods. This offers the potential for the clinical implementation of vascular nanomedicine systems. (Scheme 1).

## The classification and characteristics of vascular anomalies

### Vascular tumors

Vascular tumors are separated into benign, locally aggressive or borderline, and overtly malignant (Table 1). The most common benign vascular tumor is infantile hemangioma (IH). IH occurs in between 4 and 10% of all infants and children, and is more frequent in cervicofacial locations [1]. The natural history of IH is a distinctive growth pattern with a rapid proliferative phase followed by spontaneous involution. In the proliferative phase, hemangiomas grow quickly in three to six months after birth and then gradually regress into fibrofatty tissue in childhood [5]. However, some IH can occur permanent lesions, and about 10–15% of IH are destructive, increasing the risk of functional impairment and disfigurement [6]. Hence, the appropriate treatment is essential for IH. Hemangioma stem cells (HemSCs) and hemangioma endothelial cells (HemECs) are the most important components in the course of infantile hemangioma. The HemSCs have the potential to proliferate and disintegrate into various different cell lineages, which is one of the pathogenies of IH. The HemECs also result in a disorder of angiogenesis and further form hemangioma [7]. IHs possess kind of unique immunohistochemical markers, such as glucose transporter 1 (GLUT1), SALL4, and CD133 [7, 8], which have important roles in the diagnosis and treatment. Utilizing these specific markers, there is potential for the development of targeted nanomaterials that could revolutionize the approach to IH diagnosis and therapy. Such advancements could lead to more accurate and tailored medical treatments, enhancing personalized care in clinical settings.

**Table 1:** Overview table for ISSVA classification of vascular anomalies

Vascular anomalies				
Vascular tumors	Vascular malformations			
Benign	Simple	Combined	Of major named vessels	Associated with other anomalies
	Capillary malformations (CM)	Associate two or more vascular malformations in one lesion	Affect veins, arteries, or lymphatics of generally large caliber, often axial or conducting vessels	Syndromes in which vascular malformations are accompanied by other symptoms in addition to vascular anomalies
Locally aggressive or borderline	Lymphatic malformations (LM)			
	Venous malformations (VM)			
Malignant	Arteriovenous malformations (AVM)			
	Arteriovenous fistula (AVF)			

Locally aggressive or borderline tumors, such as Kaposiform hemangioendothelioma, can damage the skin and subcutis, and penetrate the deep tissues, presenting as a locally aggressive tumor [9]. Superficial lesions are firm, ecchymotic, purpuric, and painful. Lesions are usually unifocal and growth is contiguous. It histologically resembles tufted angioma with larger and confluent tumor lobules, with a more infiltrating pattern [10]. Kaposiform hemangioendothelioma focally expresses lymphatic endothelial markers (podoplanin, Prospero homeobox 1) which can be distinguished from hemangioma [11].

Malignant vascular tumors are rare and mainly contain angiosarcoma and epithelioid hemangioendothelioma (EHE). Angiosarcoma can arise in any part of body. More than half of angiosarcomas are cutaneous, with the most common involved area being the head and neck region, particularly the scalp [12]. Soft tissue angiosarcomas seem to be multinodular hemorrhagic tumors that frequently have subsequent cystic degeneration and necrosis [13]. The typical vascular markers of angiosarcomas generally include CD34, CD31, Fli1, ERG, and occasionally podoplanin (D2-40), a lymphatic marker [14]. EHEs are less common than angiosarcoma and have a more indolent nature. EHEs have the tendency for angiocentric growth, enlarging the vessel wall, obliterating the lumen and spreading centrifugally into surrounding tissue where they induce a sclerotic response [13].

### Vascular malformations

Vascular malformations are subclassified into four groups, including simple malformations,

combined malformations, malformations of major named vessels, and malformations associated with other anomalies (Table 1). The simple malformations are further subclassified based on the type of blood vessels with anomalies: capillary, lymphatic, venous, and arteriovenous malformations [15].

Capillary malformations (CMs) usually appear at birth persisting throughout life of the patient, and mainly affect the skin and mucosa. The CMs begin to appear as pink to red macules and become thick and dark associating with bone overgrowth or soft tissue [16]. Lymphatic malformations (LMs) are made up of differently dilated lymphatic cysts or channels bordered by lymphatic endothelial cells. Common LMs appear under normal-colored skin and mainly locate in the axillary and cervicofacial region. They are classified as microcystic, macrocystic, and mixed subtypes [17]. LMs are usually solitary or multifocal and grow slowly and involutes rarely. They can rapidly enlarge due to hemorrhage or infection, thus leading to potential compression of structures. The radiographic feature is an effective method to define the difference of LMs because of the evident fluid-filled areas of macrocystic [18]. Venous malformations (VMs) are local, congenital lesions of distorted slow-flow venous-like vessels, generally appearing as a blue skin discoloration when the lesion is superficial or as a soft subcutaneous mass. The first-line treatment for symptomatic VMs is typically sclerotherapy. A sclerosing substance such as ethanol is percutaneously injected into the lumen of the affected vessel, then leading to epithelial cell destruction and lesion shrinkage [19]. Arteriovenous malformations (AVMs) consist of defective arteries, veins, and capillaries that have direct arteriovenous connections, leading to arteriovenous shunting. AVMs is the more aggressive type of vascular malformation. AVMs progress throughout life and result in various complications including tissue destruction due to bleeding, rapid overgrowth, functional deficits, and severe deformities. The majority of current treatment methods concentrate on surgical, catheter-guided treatments, or stereotactic radiosurgery to resect, embolize, or radiate AVMs in order to minimize the hazards involved [20].

Combined vascular malformations occur when more than two vascular anomalies coexist in the same lesion. These can be simple malformations, malformations of large designated vessels, or a combination of the two. Malformations of major named vessels influence arteries, veins, or lymphatics of generally large caliber, often axial or conducting vessels including the persistence of embryonic vessels and congenital arteriovenous fistulas. Vascular malformations associated with other anomalies mainly are eponymous syndromes. The type of vascular malformations is often accompanied by anomalies of bone, soft tissue, or viscera [21].

In conclusion, the diverse types and subtypes of vascular anomalies exhibit varying clinical manifestations, growth patterns, and response to treatment. This heterogeneity makes accurate diagnosis and optimal treatment selection a daunting task. Moreover, the potential complications associated with treating vascular anomalies cannot be overlooked. The proximity of these anomalies to vital structures, such as nerves and organs, increases the risk of adverse events during interventions. Given these complexities and therapeutic challenges, there is an urgent need for advancements in both diagnostic tools and therapeutic approaches for vascular anomalies. With the rapid development of nanotechnology, such obstacles may be solved easier. For example, in diagnosis, the invention of nano-size contrast agents with specific surface modification can distinguish different types or subtypes of vascular anomalies and detect more minor vascular lesions accurately. In therapy, on the one hand, the nano-drug delivery system can obviously increase the drug concentration in target site; on the other hand, embolic agents, playing critical role in the treatment of AVMs, can provide improved efficacy, safety, and tailor-made characteristics to suit different clinical needs with the aid of nanotechnology and possesses multifunctional effect.

## Advantages of nanotechnology in vascular anomalies

The development of nanotechnology offers new possibilities for diagnosing and treating vascular anomalies. In diagnostics, nanotechnology enables highly sensitive and specific imaging techniques for vascular structure imaging. By incorporating nanoparticles (NPs) as contrast agents, NPs enhance the visualization and characterization of vascular anomalies through different imaging modalities such as MRI, CT, and ultrasonic imaging. In therapy, functionalized NPs or biomaterials can be engineered to selectively bind to and deliver therapeutic agents directly to diseased blood vessels. This targeted approach not only enhances the efficacy of the treatment but also minimizes the potential for systemic side effects. The controlled and sustained release of drugs from nanocarriers ensures optimal drug concentration at the target site, maximizing therapeutic benefits. The nano-size agents can be obtained using several types of matrixes (inorganic, metal, polymeric, lipid, surfactant) and various supra-molecular structures, such as micelles, dendrimers, vesicles (liposomes and niosomes), nanoemulsions, and nanoparticles (NPs) [22]. Factors like size, conformation, non-covalent interactions, and surface adsorption significantly influence the interaction between the nanocarrier and the biological environment.

In both therapeutic and diagnostic applications, nanosized agents must first be delivered into the vascular lesion area, and then drugs diffuse to have effect. Consequently, researchers are actively pursuing the development of various targeting strategies aiming to improve cargo delivery systems by enhancing their specificity and efficacy. Two targeting strategies, passive and active targeting, have been widely used to enhance the accumulation of nanosized agents in the vascular lesion tissue via systemic circulation [22]. In passive targeting, the enhanced permeability and retention effect (EPR) mediated nano-drug delivery system has been considered to be the greatest breakthrough compared to individual soluble molecules or drugs. The EPR effect theory contributes to NPs with the size range of 20–200 nm preferentially concentrating on vascular lesion sites because of defective vascular structure. Compared to healthy vascular tissue with regular and tightly arranged, the accumulation of nanoparticles is much more difficult. Due to this, during the distribution phase, nanomaterials accumulate more evident in vascular defect area and have a high permeability [23]. Hence, size is vital for the design and synthesis of nano delivery systems.

In addition to the passive targeting strategy, active targeting is considered a more effective method to obtain better specificity and affinity for vascular lesions and is easier to modify for NPs. Active targeting primarily depends on the specific interaction between modifications on NPs and certain unique receptors involved in tumor progression, invasion, and prognosis, which are expressed on the surface of the affected area. A wide range of targeting ligands as the surface modification of nanoparticles, including proteins (antibody or antibody fragments), peptides (arginine–glycine–aspartic acid or RGD), vitamin (folic acid), nucleic acid (aptamer) and glycoprotein (transferrin) are recently being investigated for the various vascular related diseases extensively [24–26]. Through delicate design and modification, nanocarriers can vastly increase drug accumulation in target lesion area by connecting ligands to receptors. Moreover, the specific surface modification of contrast agents can assist to distinguish different types vascular abnormalities. The relatively vascular surface modification can enhance their binding capability to the vascular structure. This not only contributes to detect minor lesions but also improves contrast with surrounding soft and hard tissues. In addition, surface modification significantly decreases the residue of drugs or contrast agents in normal tissues, thereby enhancing safety of treatment and imaging. Moreover, the primary mechanism for NPs clearance involves opsonization and phagocytosis by macrophages, following by receptor-mediated endocytosis [27]. To delay degradation and extend circulation period of NPs, the surface of NP can be modified with a biocompatible and non-immunogenic target group, which helps avoid degradation by the reticuloendothelial system [28].

## Nanotechnology applying in diagnosis of vascular anomalies

Vascular anomalies can be imaged and distinguished by utilizing multiple modalities, which remain a prerequisite for precision treatment. In line with the advancement of imaging modalities, there is a growing array of small molecules or molecular complexes that serve as tracer or contrast agents. These contrast agents or imaging tracers are utilized to enhance the conventional imaging modality, enabling a more distinct visualization of anomalies and facilitating the diagnosis of previously imperceptible pathologies. The efficiency of imaging agents is further enhanced by being integrated into nanosized materials, with the aim of continuously improved sensitivity, stability, and plasma residence times. We also summarized the advantages and disadvantages of different imaging modalities applying in diagnosis of vascular anomalies (Table 2).

**Table 2:** The advantages and disadvantages of different imaging methods

Imaging methods	Advantages	Disadvantages
MRI	Provide excellent spatial resolution and soft tissue contrast; visualize of intricate anatomical details; without ionizing radiation damage	Expensive; excluded patients with magnetic metal
CT	Provide precise anatomical layer information; good imaging capability for hard tissues	Low soft tissue resolution; ionizing radiation damage
Ultrasound	Provide blood flow velocity information; Good penetrative ability; high safety	Hard to image vascular structure; unable to provide functional imaging; low image contrast
PAI	Provide functional information; High resolution and high contrast; deep-tissue optical ability	Strong dependence on tissue optical properties; safety of contrast agents
DSA	Provide precise vascular structure and branches; assess blood flow dynamics; Evaluate interventional procedures effectiveness	Invasive; large amount of contrast agents

### Magnetic resonance imaging (MRI)

Magnetic resonance imaging (MRI) is a highly advantageous technique in the identification of different vascular anomalies. MRI can provide excellent spatial resolution and enhanced soft tissue contrast to visualize intricate anatomical details. The majority of vascular anomalies exhibits similar signal intensity to muscle on T1-weighted imaging (T1-WI) and increases signal intensity on T2-weighted imaging (T2-WI), with varying degrees of contrast enhancement depending on the specific abnormality [3, 29]. However, MRI with relatively low sensitivity may be difficult to distinguish the subtle and complex lesions or anomalies. Researchers proven that paramagnetic complexes such as gadolinium (Gd^3+^) or manganese (Mn^2+^) ions can improve the MRI signal by accelerating the T1 relaxation rate of surrounding water protons. This results in a brighter signal to improve the signal-to-noise ratio (SNR) in MRI imaging [30, 31]. Hence, the synthesis and design of Gd (III) complexes are crucial for enhancing the efficiency and effectiveness of MRI contrast agents. Researchers have explored various strategies, including attachment of Gd (III) complexes onto dendrimers/dendrons to increase the relaxivity and stability [32, 33]. Indeed, the conventional method of preparing dendrimer/dendron-based MRI contrast agents by integrating small molecule Gd (III) complexes onto the surface of dendrimers/dendrons always cause a waste of the internal skeleton and a limited increase in T1 relaxivity [34, 35]. Based on that, fu et al. investigated branched polymer-based MRI contrast agents via simple “Ax + By”-type one-pot polymerization to attain poly-DOTA branched polymers with DOTA as the internal skeleton. This Gd branched polymers not only possessed the similar structural advantages as dendrimers/dendrons for MRI imaging, but also simplified tedious and synthesis procedure of dendrimers/dendrons. This branched polymer-based Gd complex exhibited higher kinetic stability, T1 relaxivity, and good biosafety for vascular structure imaging. The prolonged observation window also provided an opportunity to detect and diagnose complicated vascular disease. Moreover, most of branched polymer-based Gd complex could be excreted from the body through liver and kidney metabolism for a few days, avoiding the risk of organ toxicity caused by long term retention of Gd (III) in the body [36].

Furthermore, the progress of nanotechnology has provided valuable opportunities for utilizing nano- and micro-sized systems in MRI. Imaging nano-systems, characterized by their specific size and surface properties, enable a deeper detection of the unique features of neoangiogenic vasculature. This is particularly useful in assessing angiogenesis by measuring alterations in vascular volume and permeability [37]. Hence, Gd-based NPs can achieve better contrast imaging ability and lead to a considerable improvement in relaxivity compared to small molecular Gd-chelates [38]. For instance, Gd-loaded apoferritin system, serving as an imaging contrast, could target newly formed endothelial vessels in vivo. Despite each protein only contained ten Gd complexes/protein in the internal cavity, the system exhibited high efficiency due to its remarkable relaxivity. As a result, a lower dose of Gd, specifically 0.01 mmol/kg, could still achieve the desired imaging outcomes. By using a lower Gd dose, the risk of potential short- and long-term toxicity associated with higher doses could be reduced [37].

**Fig. 1:**
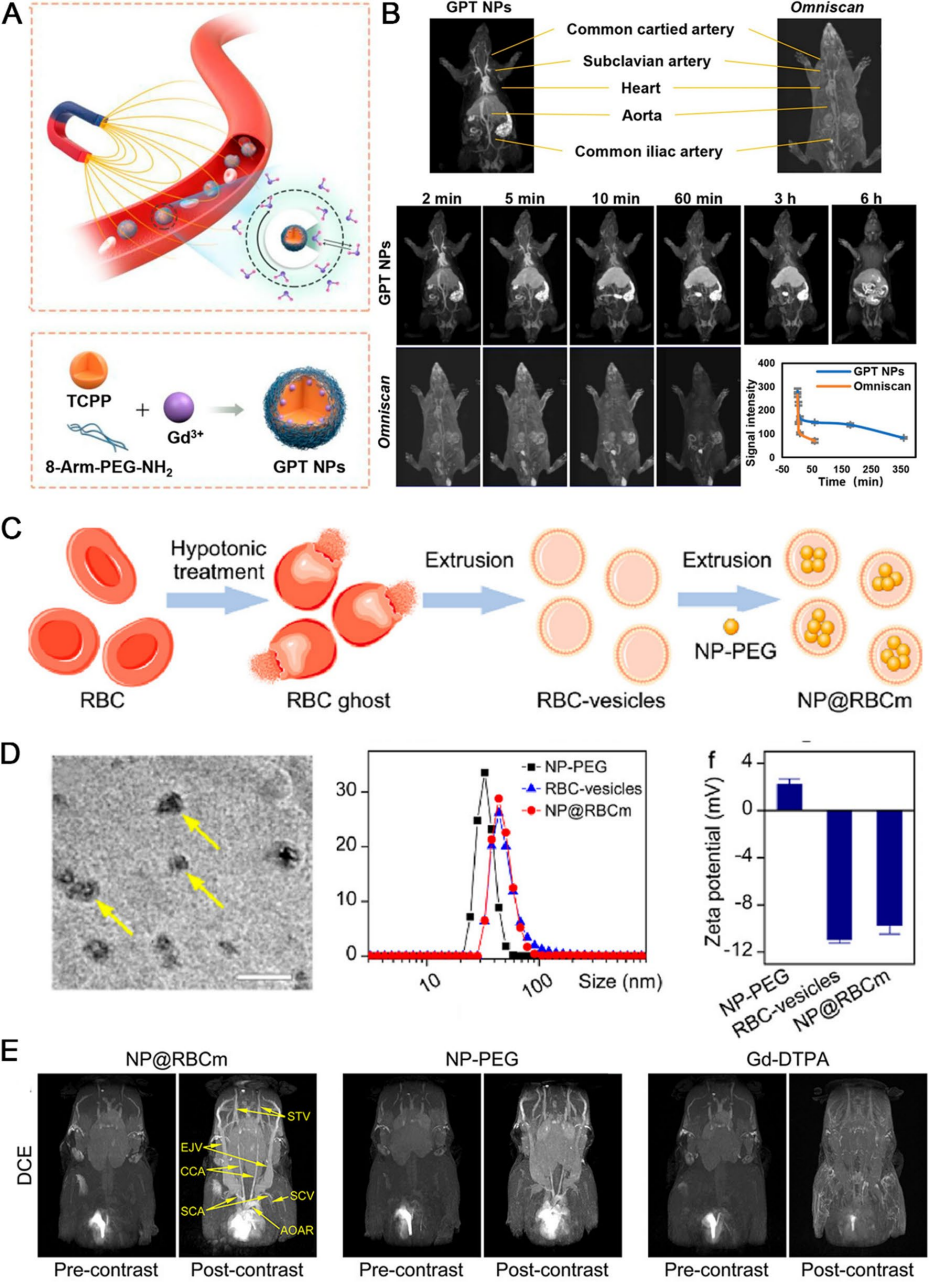
MRI based on Gd^3+^ nanoplatforms. **A** A schematic diagram illustrates the facile synthesis of Gd-chelated TCPP PEG nanoparticles (GPT NPs) of the preparation process of the NP@RBCm nanoprobes. **B** Vascular imaging performance of GPT NPs in rats compared with traditional MRI contrast agent (Omniscan). Copyright 2022, Springe nature. **C**: Schematic of the preparation process of the NP@RBCm nanoprobes. **D**: The characteristics of NP@RBCm nanoprobes; **E**: MRI angiography in vivo. Copyright 2022, American Chemical Society

Except for the protein carrier, the organic nanoplatforms were also utilized to encapsulate Gd complex. The tetrakis (4-carboxyphenyl)-porphyrin (TCPP) and 8-arm-amine-polyethylene glycol (PEG) were used to modify chelated­ Gd^3+^ (Gd-chelated PEG-TCPP nanoparticles, GPT NPs). The core of TCPP provided a stable environment for Gd^3+^ coordination, enhancing its paramagnetic properties, while the outer surface PEG given GPT NPs extended blood circulation time and superior biocompatibility. MRI imaging in vivo demonstrated that these GPT NPs offered higher-resolution arterial visualization compared with clinically used Gd-based contrast agents, displaying a clear arterial image with minimal overlap with enhancing veins and tissue [39] (Fig. 1A, B). In order to avoid the immune clearance caused by “protein corona” of NPs, he et al. developed a biomimetic approach by encapsulating ultrasmall NaGdF_4_ nanoparticles into the red blood cell membrane (RBCm). The coated RBC membrane gave the nanoprobes a stealthy quality within the circulatory system, supplying a longer time window for long-term vascular surveillance. And this biomimetic NPs with outstanding MRI angiography ability could observe most main arteries and veins, and even the microvessels of mice, which could be greatly used for high-resolution three-dimensional dynamic contrast enhanced MRI angiography and vascular disorders in an extended time window [40] (Fig. 1C–E).

The diameter of lymphatic vessels is relatively small, not much larger than the diameter of a syringe needle, making the injection of MRI contrast agents quite complex and challenging. Therefore, the size of the nano-contrast agents becomes a crucial factor for compatibility with the lymphatic system. Yano et al. encapsulated cyclic-chained Gd-based contrast agents in carboxylated nanodiamond (CND) particles for targeted imaging of lymphatic systems. The DOTA-based CND contrast agents possessed exceedingly small diameters ranging from 3 to 10 nm in distilled water and serum, indicating that these NPs could be selectively endocytosed by lymphatic vessels and effectively filtered in the kidney for final excretion to avoid toxicity. Therefore, this newly fabricated DOTA-based CND contrast agents (Gd-DOTA-CND) could be promising alternatives of Gd-chelates for the selective MR lymphatic imaging [41].

The other important NPs for MRI imaging are iron oxide particles. These particles are widely utilized in MR molecular imaging applications due to their remarkable efficiency in reducing signal intensity in T2*-weighted images, enabling the imaging of molecular targets even at very low concentrations of particles. The specific diameter of iron oxide particles named Superparamagnetic iron oxide-SPIONs (less than 30 nm) have superparamagnetism under magnetic fields. However, SPIONs are easily eliminated from the circulation by the reticuloendothelial system in vivo. This limitation can be mitigated by employing specific coating biomaterials to enhance the stability and circulation time. Moreover, combined with specific ligand modifications, the iron oxide particles can be easily accumulated in the specific vascular lesion. Sohn et al. conjugated Fe_3_O_4_ NPs with anti-GLUT1 antibody as a targeting moiety and applied in differentiating infantile hemangioma from vascular malformation [42]. These anti-GLUT1 antibody-Fe_3_O_4_ NPs resulted in a significant increase uptake by human umbilical vein epithelium and accumulated obviously in mouse model of hemangioma in vivo, holding great promise in distinguish different vascular anomalies.

Above all, the MRI NPs mainly focus on T1-shortening or T2-shortening mechanism to generate either positive or negative MRI signal contrast, respectively. The application of nanotechnology assists the contrast agents to prolong blood circulation in vivo. Utilizing targeting strategies not only can help to distinct different lesions but also guides contrast agents to specific sites where the local contrast will be strongly enhanced.

### Computed tomography (CT)

X-ray-based techniques including computed tomography (CT), are among the most widely used and the oldest clinical imaging modalities. However, in contrast to MRI and ultrasound, CT has limited use in the imaging evaluation of vascular malformations because of concerns about exposure to ionizing radiation and low soft tissue resolution. Hence, CT is more suitable for imaging vascular tumors [43]. The most urgent problem to be solved in CT imaging is the risk of exposure to harmful ionizing radiation, which can cause damage to adolescents and teens. CT imaging is based on the accumulation at high concentrations required to overcome the detection threshold. Consequently, the strategies of CT-based NPs are mainly focused on enhancing accumulation to increase contrast sensitivity and decreasing radiation.

Iodine has been the most widely used X-ray contrast agent in clinical CT imaging. Due to the low molecular weights, the iodine agents can be quickly removed from the blood via renal filtration with approximately half of the iodine cleared within 45 s. The short window of time to perform an image often results in precluding appropriate clinical diagnosis [44]. In order to prolong blood half-lives and enhance specific targeting capability, the iodin agents can be encapsulated into various nanocarriers, including liposomes, nano-emulsions, liposomes emulsions with chylomicrons, micelles, dendrimers [45–47]. However, the reduced content of some nanosized iodine contrast agents requires a larger dose and high concentration for CT imaging in vivo, remaining a challenge in the clinical application. Hence, investigating methods to enhance the concentration in the imaging area is the aim in the design of iodine-based nanosized contrast agents. Zou et al. developed poly (ethylene glycol)-b-poly (iodine trimethylene carbonate) (PEG-b-PIC) nanopolymersomes possessing an ultrahigh iodine content for high-performance in CT imaging. These radiopaque nanopolymersomes exhibited excellent colloidal biocompatibility and stability, low viscosity, iso-osmolality, with a small size. The in vivo imaging demonstrated that they had a prolonged circulation time and could be used for blood pool as well as reticuloendothelial system imaging over a few hours [48].

Currently, gold nanoparticles (AuNPs) have increased use as contrast agents for CT imaging of blood pools [49]. Compared with iodine agents, due to its higher electron density and atomic number, gold has a higher robust capacity for light scattering and photon absorption, with a greater ability to enhance contrast on CT. Moreover, AuNPs have better biocompatibility, and the surface of AuNPs can be easily modified with various functional groups, such as targeting molecules or specific biomarkers, bestowing properties upon the final particles that are advantageous for a variety of applications [50]. For instance, the PEG-coated colloidal AuNPs had the capacity to penetrate tumors and visualize vascular structures. Radioactive iodine-labeled, cyclic RGD-PEGylated AuNPs probes (^25^I-cRP-AuNPs) targeting αvβ3 integrin specifically had efficient and long-term tumor-targeted imaging capability with almost no cytotoxicity [51]. AuNPs that were EGFR-targeted allowed for efficient CT visualization of the tumor-associated vasculature in mouse head and neck carcinomas. These tumors are quite small, measuring just 4–5 mm [52]. Notably, AuNPs have a lot of advantages in CT visual imaging, but gold particles are non-degradable, and hard to clear in vivo. Moreover, the challenge remains, as the toxicity of AuNPs in vivo is still not well understood. There is still a long way to be translated into clinics for AuNPs successfully.

### Ultrasound

Ultrasound (US)-based imaging is a preferred modality for detecting and diagnosing vascular anomalies. US can assess the extent of the disease and the degree of vascularity, without the use of ionizing radiation [53]. When vascular malformations are still in the differential diagnosis, US is commonly used as the initial modality for examining newly skin lesions or protuberant lesions. It is also very useful for monitoring and assessing the effectiveness of lesion therapy. Doppler ultrasound using spectral and color information help distinguish between vascular anomalies with high and low flow [3].

Microbubbles (MBs) have been widely used as the enhanced ultrasonic contrast agents and applied in the clinical diagnosis successfully. The MBs are usually surrounded by a phospholipid, protein or biodegradable polymeric shell structure to stabilize the formulation and filled by gas core (air, perfluoropropane, or hexafluoride). Nevertheless, the majority of MBs have a diameter of 1–8 μm, which severely restricts their use in vasculature imaging [54]. The MBs are difficult to penetrate through the leaky blood vessel walls resulting in a short retention time, and thus it is crucial to reduce the size of the echogenic bubbles to the nanoscale without reducing visibility on ultrasound. To overcome such limitation, nanoscale ultrasonic contrast agents (nano-UCAs) has been extensively and deeply explored, due to advancements in nanotechnology and biomaterials. Nano-UCAs primarily consist of polymer NPs, liposomes, metal NPs and metal oxides, nanobubbles, nanodroplets, as well as carbon-based materials [55]. The smaller size of the nano-UCAs can enhance accumulation at target sites through EPR effect. Furthermore, it is much easier to attach additional extravascular targeting components to the surface of nano-UCAs. Moreover, due to the large internal volume and specific surface areas, a wide range of imaging agents or drugs can be simultaneously loaded into these nanomaterials using simple techniques.

**Fig. 2:**
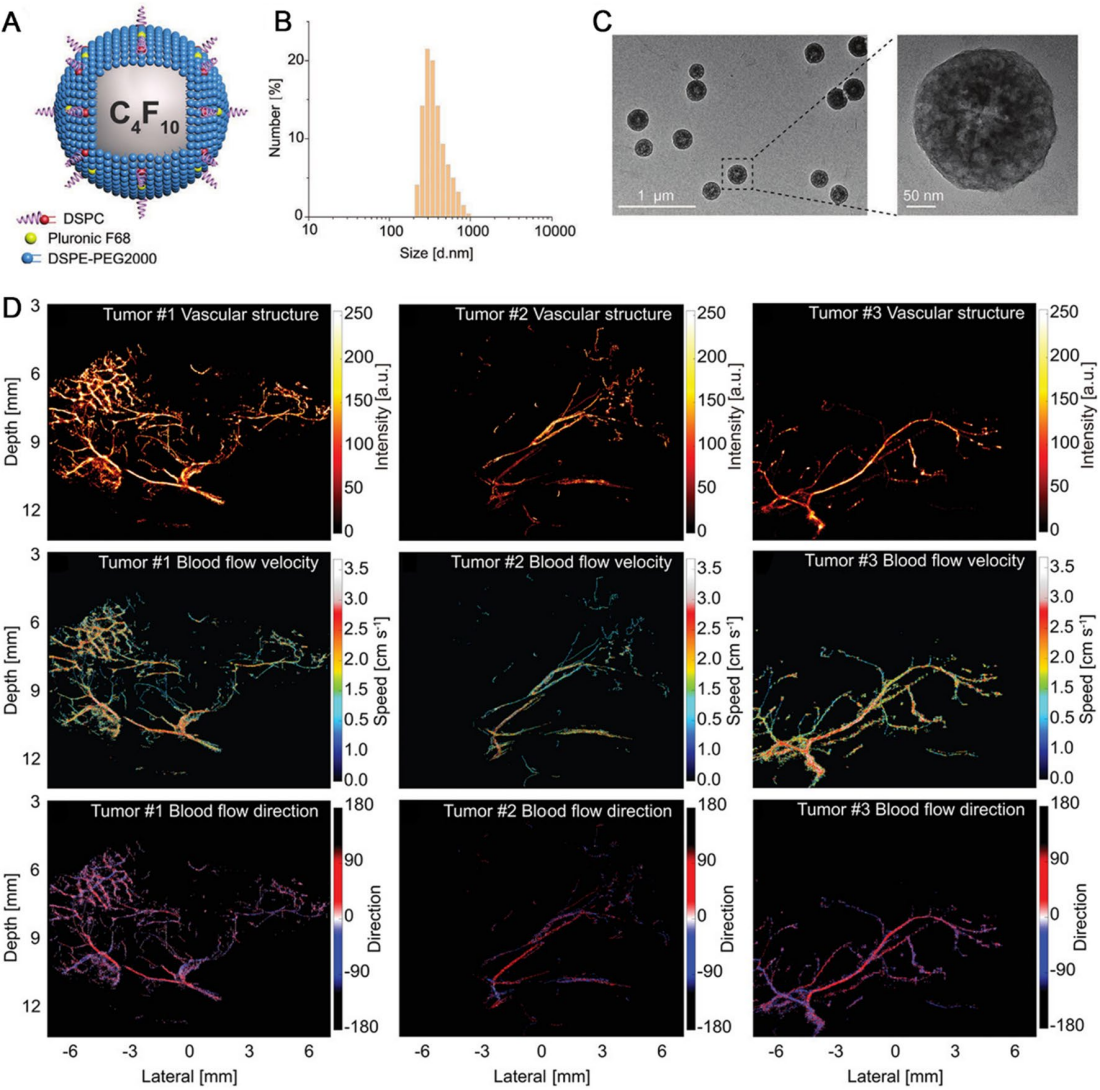
US imaging agents shown vascular structure. **A** The schematic core–shell structure of the PCND. **B** PCND size distributions are characterized by dynamic light scattering (DLS) method; **C** TEM of PCNDs. **D** ALUSA imaging of vasculature and hemodynamics in tumors; up: vascular structures, middle: the distribution of the corresponding blood flow velocity. Down: the distribution of the corresponding blood flow direction. Copyright 2022, Wiley

To allow for particle aggregation at the targeted region, the ideal nano-UCAs should have a suitable circulation half-life, ranging from several days for therapeutic applications to hours for imaging applications. The agents also must be stable in solution (i.e., resist aggregation) and prevent reticuloendothelial absorption [56]. The additional extravascular targeting moieties on the surface of UCAs can further enhance their accumulation and retention. For instance, the nanobubbles can be modified with molecular markers of angiogenesis such as vascular endothelial growth factor receptor type 2 (VEGFR 2) to enhance contrast ability [57]. Although, the enhanced US contrast agents possess higher resolution and imaging clarity to observe internal structure of tissues and organs. However, the US imaging is hard to observe the perfusion of a single blood vessel, as well as its blood supplies to the downstream vessels. To solve such situation, an et al. fabricated a new perfluorobutane (PFC) nanodroplet through spontaneous droplet nucleation and developed an arterial labeling US subtraction angiography (ALUSA) based on proposed phase-change nanodroplets (PCNDs). The PCNDs were composed of three components: a core to reduce the activation threshold, a shell made of the phospholipids DSPC and DSPE-PEG2000 to stabilize the newly converted gaseous microbubble, and a surfactant pluronic F68 to control surface tension. This synthesized PCNDs provided color-coded super-resolution ALUSA image just like digital subtraction angiography, exhibiting the downstream arcuate and interlobular arteries of each segmental renal artery in a rabbit kidney. ALUSA could also offer the vascular structures, blood flow velocity with a resolution well beyond the wave diffraction limit. The novel nanodroplets had a potential for US imaging to diagnose and assess vascular-related diseases (Fig. 2) [58].

Although currently technology and software for therapeutics-mediated ultrasonic release at the target site have an evident progress, there still need to be improved specifically for nano-UCAs. It would be ideal to design transducers that can deploy and image simultaneously in three dimensions. On the formulation front, it is crucial to continue developing focused, tiny, highly echogenic formulations that offer extended stability and prolonged circulation times. Molecular imaging with ultrasound is expected to find widespread applications in the diagnosis of vascular anomalies in the future.

### Photoacoustic imaging (PAI)

Photoacoustic imaging (PAI) can obtain superior imaging by transforming optical signals into acoustic signals for image formation. When pulsed laser light irradiates biological tissue, it quickly absorbs laser energy, causing tissue to expand from warmth and then produce ultrasonic waves. These waves, referred to as photoacoustic (PA) signals, are then captured by an ultrasonic transducer [59–61]. Researchers have developed an array of configurations for imaging across multiple scales, ranging from micro-level, encompassing organelles and cells, to meso- and macro-scales, which include tissues and entire organs. These systems are crafted by integrating diverse methodologies for optical illumination and acoustic signal detection [62]. Significantly, PAI has attained a fine spatial resolution at the micron level alongside delivering satisfactory image quality for vascular structures [63]. PAI also has functional capability to provide detailed information, such as blood oxygen, blood flow and temperature of local lesion, to assist in the diagnosis of different kinds of vascular anomalies [64]. Moreover, the preprocessing of images is facile. Therefore, PAI signals possess both optical and acoustic characteristics as well as imaging abilities, well-suited for vascular visualization.

Endogenous contrast agents for PAI are intrinsic chromophores in biological tissues, primarily including hemoglobin, lipids, melanin, bilirubin, collagen, water, DNA, RNA, etc [64–67]. Molecular-level changes during the development of vascular anomalies result in variations in photoacoustic signals from these molecules, forming the basis for the use of endogenous contrast agents. Despite the greater biological safety of endogenous contrast agents to decrease risk, the endogenous contrast agents lack specificity or sensitivity [68]. Hence, researchers have been devoted to various exogenous contrast agents for PAI, mainly including organic dyes, genetically encoded proteins and micro/nanoparticles [69].

Specifically, the nanoparticles primarily consisted of metals, carbon and organic polymer NPs. Current research on metal particles focuses particularly on synthesizing gold nanoparticles of diverse geometries, mainly comprising gold nanorods (AuNRs), gold nanospheres (AuNS), gold nanocages (AuNCs), and other shapes. Hollow gold nanostructures have also been particularly noted for their potential applications in drug delivery [70]. However, the toxicity of mental NPs cannot be ignored. Carbon nanomaterials have demonstrated superior performance over gold nanomaterials in terms of photostability, biocompatibility and toxicity, as well as fabrication [71]. Furthermore, the semiconducting polymer nanoparticles (SPNs) were synthesized from bioinert materials. Pu et al. have proved that SPNs possessed high structural flexibility, narrow photoacoustic spectral profiles and strong resistance to photodegradation and oxidation, indicating that SPNs could be utilized as an ideal nanoplatform for developing photoacoustic molecular probes [72].

Recently, the activatable nanoprobes for PAI have been extensively explored in the PAI. Activatable nanoprobes are designed to be responsive only at sites of lesions, where they can be triggered by disease-specific small molecules or the unique microenvironment of the disease. Such activatable imaging probes significantly reduce background noise signal and enhance imaging sensitivity and specificity, which is particularly vital for accurate detection of minute lesions [73]. As we all know, the development of vascular tumors (e.g., hemangiomas) have a tight connection with proliferative changes of endothelial cells. In this phase, the GSH level, an important component in altering the oxidation–reduction status, is obviously increased. Therefore, developing active PAI probes based on GSH may assist to image specific vascular tumors [74]. Gong et al. conducted research using MnMoOX nanorods to track glutathione (GSH). This process involved reduction of Mo (VI) to Mo (V0), transforming nanorods to NPs and increasing PAI signal for tumor-specific imaging [75]. Besides, hypoxia is also a critical index in excessive angiogenesis and can help evaluate the progression of vascular lesion. Contrast agents can also bind to upregulated endogenous biomarkers in tumor hypoxia site. The organic dye hyP-1, derived from Hypericum perforatum, can undergo bio-reduction by heme proteins such as cytochromes P450 (CYP450), transforming into “red-HyP-1”. This allows for the assessment of hypoxia degree in different areas of the tumor based on the signal intensity [76].

In conclusion, PAI, a non-ionizing and radiation-free imaging technique, has garnered growing attention among researchers. It not only provides qualitative information about diseases but also holds promise for theranostic applications. Therefore, PAI is expected to evolve into a potential diagnostic imaging tool and contribute to vascular disease treatment.

### Digital subtraction angiography

Digital subtraction angiography (DSA) is a diagnostic imaging technique used to assess vascular diseases, especially in the high-flow vascular anomalies [77]. It involves the injection of a contrast agent into the bloodstream to highlight the blood vessels on X-ray images. During the procedure, a series of X-ray images are taken before and after the injection of the contrast agent. By subtracting the pre-contrast images from the post-contrast images, background structures are eliminated, allowing for a clearer visualization of the blood vessels. DSA is particularly valuable for detecting abnormalities such as stenosis, aneurysms, arteriovenous malformations, and vessel blockages [78]. The real-time nature of DSA enables assessment of blood flow dynamics and evaluation of effectiveness of interventional procedures, such as embolization therapy. This provides clinicians with crucial information for making accurate diagnoses and guiding therapeutic interventions. However, DSA requires higher concentrations of X-ray contrast agents due to lower X-ray energy and resolution [79]. The use of a large amount of contrast agents will obviously increase the risk of contrast-induced nephropathy. Similarly, AuNPs play an important role in DSA imaging; however, their heavy aggregation at high concentrations greatly limits their application due to insufficient stability. In order to improve the colloid stability of AuNPs, some hydrophilic macromolecules, such as PEG and dendrimers, have been synthesized for the surface modification of AuNPs. These modified AuNPs have shown great potential as CT contrast agents for blood-pool imaging [80]. Consequently, there is a pressing need for a noninvasive labeling and high-resolution tracking technique that utilizes non-toxic contrast agents. This technique would allow for the visualization of individual blood vessels and their subsequent perfusion in a safe and effective manner.

### Others

Despite the various imaging modalities for vascular anomalies in vivo, the application of nanomaterials in the field of in vitro diagnostics has also attracted increasing attention. Detecting the biomarkers of different diseases offers a non-invasive, convenient method that assists in distinguishing of various types of diseases, thus holding great potential for early diagnosis, rapid testing, and point-of-care testing [81]. In recent years, nano-biosensors combining with nanoprobes has emerged as an alternative to traditional technologies, offering potential advantages including high sensitivity, selectivity, accuracy, cost-effectiveness, rapid detection, stability, accessibility, and ease of use [82]. Biosensors utilize a range of nanoprobes as biometric recognition components, which are immobilized on the sensor surface. These nanoprobes then transformed into measurable electronic or optical signals via biological interactions with the target substance. For example, to help recognize locally aggressive or borderline and overly malignant vascular tumors, some vascular relative biomarkers, such as carcinoembryonic antigen (CEA), human epidermal growth factor receptor 2 (HER 2), vascular endothelial growth factor 165 (VEGF 165), microRNAs [83], can be designed as biomarker nanoprobes of nano-biosensors to accelerated the process of malignant vascular tumors diagnosis, and obtain higher sensitivity and faster screening. Li et al. proposed a sandwich-type electrochemical biosensor to detect CEA. The cerium-based metal–organic framework (MOF) was synthesized and integrated with silver nanoparticles (AgNPs) and horseradish peroxidase to catalyze H_2_O_2_, consequently amplifying the current signal. This immune sensor demonstrated excellent detection capability, even capable of detecting CEA at levels below 0.2 pg/mL [84].

However, despite many progresses have been developed in diagnosis approaches by nano-biosensors or nanoprobes. The clinical application of nano-biosensors or nanoprobes are still limited. The clinical translation of nano-biosensors still faces some challenges. Furthermore, sensors specifically designed for detecting vascular anomalies should also receive more attention from researchers.

## Nanotechnology applying in therapy of vascular anomalies

Current therapeutic options of vascular and lymphatic malformations mainly include sclerotherapy, embolization, medical management, surgical intervention, laser ablation, or combination of these modalities [3]. However, for large and extensive vascular malformations, achieving complete cures remains highly challenging. To address the limitations associated with traditional therapeutic agents and approaches, the rapid advancement of biomaterials and nanotechnology in the biomedical field offers promising opportunities. In this perspective, we highlighted the emerging and innovative applications of biomaterials and nanotechnology for the treatment of vascular anomalies, aiming to overcome these limitations. These advancements hold the potential to provide more targeted, controlled, and effective therapies for patients with vascular and lymphatic malformations.

### Embolization and sclerotherapy

**Fig. 3:**
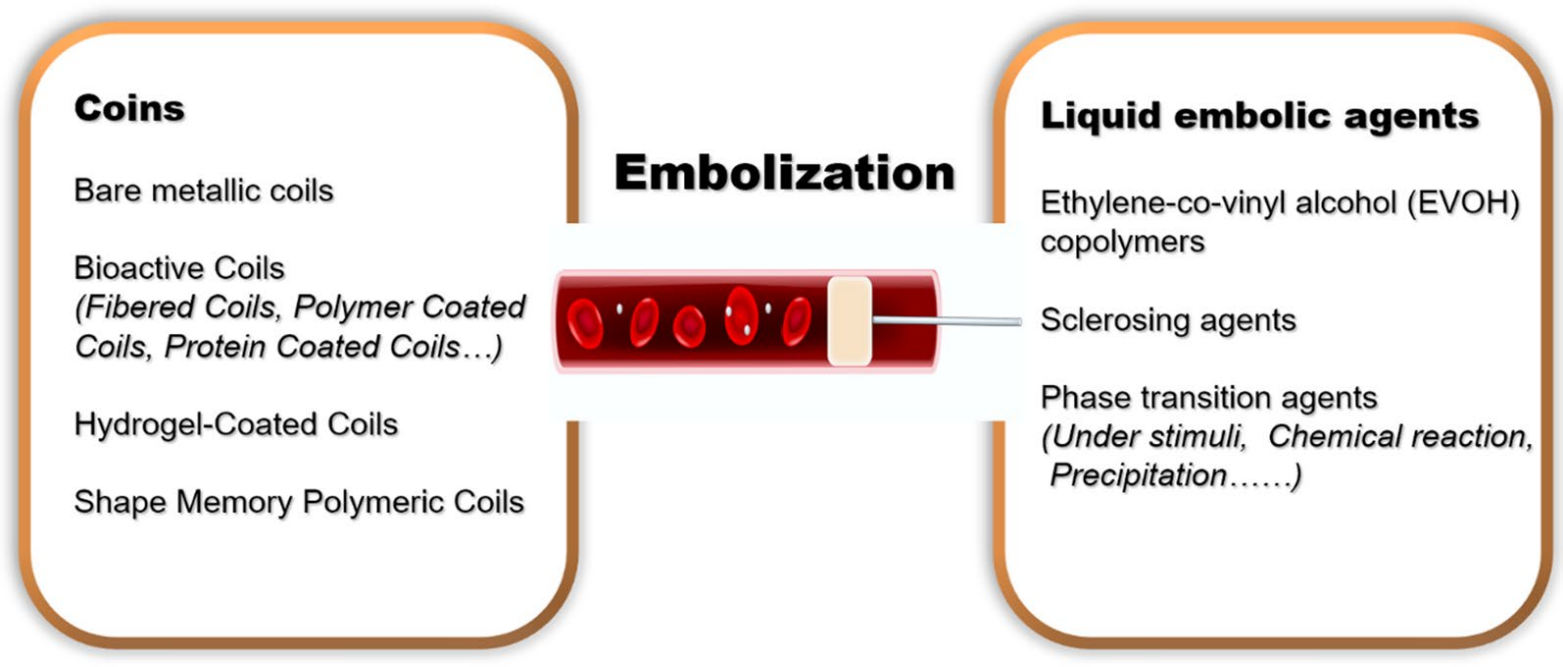
Summary of common clinical embolic agents applying in vascular diseases

Embolization and sclerotherapy are crucial treatment modalities for vascular malformations, particularly AVMs and VMs [3, 85]. In comparison to surgical resection, embolization sclerotherapy offers many advantages, such as a lower incidence of cosmetic disfigurement, minimal external scarring, ability to aid in confirming the lesion, and reducing bleeding before surgery [86]. Embolization is a noninvasive medical procedure that intentionally blocks the bloodstream by delivering emboli into the blood vessel, which can stop and prevent bleeding or cuting off the abnormal connections between arteries and veins to reduce the size of lesions. This noninvasive medical procedure usually requires guidance of an imaging system to accurately visualize the abnormal vascular structures, to ensure precise treatment and procedural safety. More importantly, embolic agents have a significant impact on the efficacy of treatments and play a significant role in developing transcatheter arterial embolization (TAE) techniques [87]. Particularly for high-flow malformations like AVMs and fistulas, embolic agents must withstand arterial high pressure and remain securely in place. Once they migrate throughout the body and finally reach the heart via draining vein, it may lead to severe complications and even seriously affect safety of patients. Hence, ideal embolic materials need to meet various requirements, including excellent imaging capabilities, efficient embolization of blood vessels, optimal flowability through catheters, good biocompatibility, and high resolution in angiography [88]. Commonly utilized embolization techniques for vascular anomalies mainly involve detachable coils and liquid embolic agents, which provide precise control during the procedure [89, 90]. These techniques help achieve optimal outcomes in the embolization of vascular anomalies (Fig. 3).

The traditional coils primarily have bio-inert metallic cores and contain coating. In theory, the metallic cores in embolization should have a higher longitudinal strength while exerting lower radial forces to minimize stress on the vessel wall [91]. These cores are often made of dense metals, which are inherently radiopaque, thus facilitating fluoroscopically guided and targeted embolization procedures. The primary mechanism of metallic coil embolization involves mechanical occlusion with minimal thrombogenic properties. To further enhance thrombus formation, researchers have developed coils with various bioactive coatings. The bioactive coatings of coins with surface modification contribute to promoting tissue response and thrombus formation, reducing recanalization and recurrence, and enhancing biocompatibility. The coating agents mainly include polymer, protein, and hydrogel, each possessing unique properties suitable for various vascular diseases [87]. These coating materials serve to enhance the performance and efficacy of embolic agents in specific clinical applications. Recently, the shape memory polymeric coils can be delivered in a temporarily compressed form through a minimally invasive approach and can then be expanded into a permanent shape in response to external stimulation.

In comparison to coil embolization, liquid embolic materials have the capability to penetrate into smaller vessels to be homogeneously filled and limit risks of recanalization. The application of liquid embolic agents also provides enhanced controllability during procedures. The primary liquid embolic materials used clinically for the treatment of vascular lesions include ethylene-co-vinyl alcohol (EVOH) copolymers and sclerosing agents [92, 93]. These agents are crucial in ensuring effective embolization and have demonstrated positive outcomes in managing vascular anomalies. EVOH copolymers mixed with dimethyl-sulfoxide (DMSO) and micronized tantalum powder for radiopacity are permanent when enter into the vessels [94]. DMSO quickly diffuses into the blood after being injected, and then EVOH precipitates capturing the tantalum powder. Over a few minutes, EVOH polymer gradually undergoes gelation. This gelation process takes approximately 10 min, enabling the operator to control injection amount and rate while monitoring the shape of the emboli in real-time using X-ray fluoroscopy. However, the major limitation of EVOH copolymers is the application of the DMSO, DMSO has both systemic and local cardiovascular toxicity. Hence, a slow injection rate (0.16 mL/min) is crucial to prevent vasospasm and vascular toxicity [95]. Besides, specific syringes and catheters that are compatible with DMSO are also required due to its organic solubility.

Sclerosing agents, such as sodium tetradecyl sulfate (STS), doxycycline, bleomycin, and ethanol, are liquid drugs that specifically target and damage blood vessels, primarily the endothelium, leading to the eventual occlusion of vascular lesions [96]. Among these, ethanol is the most frequently used in clinic and is associated with the lowest recurrence rates for vascular malformations [97]. In the therapy of AVMs, the maximum tolerated dose for absolute ethanol injection is 1 mL/ kg to a maximum of 15 per embolization procedure [98]. Administration of ethanol beyond the tolerated dose can lead to severe and potentially fatal adverse events, including seizures, respiratory complications, and cardiac arrhythmias. Due to the rapid diffusion from arteries to the capillaries level, special attention is necessary when using ethanol in proximity to the skin to prevent the occurrence of skin necrosis. Meanwhile, it usually combines with coils in the embolization of high-flow arteriovenous. Hence, it is necessary to develop more safe and stable embolic agents.

With the advancement of biomaterials and nanotechnology, an increasing number of embolic agents have been developed. These new materials offer promising alternatives for embolization procedures and are designed to provide improved efficacy, safety, and tailor-made characteristics to suit different clinical needs. Liquid-to-solid phase transitions technique has been widely investigated and developed to overcome limitations of traditional embolic materials. Regarding the mechanism of liquid-to-solid phase transition, they may be divided into three basic categories: phase transition under stimuli, chemical crosslinking between monomers, and precipitation by efflux of carrier solvent [99]. The biomaterials of phase transition under stimuli are the most commonly used and well-suited for the treatment of AVMs. The phase transition embolic agents can alter their physical states when exposed to certain stimuli, such as changes in temperature, pH, or sudden concentrations of particular ions, phase-transitional. This kind of liquid embolic agents may be favorable in terms of controllability and sensitivity because phase transition depends on particular environmental conditions [100].

Ploy(*N*-isopropylacryla-mide-*co*-butylmethacrylate) (pNIPAM)-based nanogels possess temperature sensitivity and have been widely investigated for the embolization of AVMs [101]. pNIPAAm-based nanogels consist of crosslinked polymer nanoparticles that are highly hydrophilic, allowing them to hold substantial amounts of water without dissolving [102, 103]. Moreover, the various cargos (nanoparticles, drugs, imaging agents) can be encapsulated into polymeric hydrogels and delivered simultaneously to vascular lesions carrying out multiple functions [102]. Based on pNIPAAm-based embolic agents, nanometer-sized gels (PIB nanogels) could be synthesized via precipitation–emulsion polymerization, which can improve hydrogen-bonding interactions of radiopaque agents and stabilize lower critical solution temperature of the polymers [104, 105]. Ma et al. utilized PIB nanogels encapsulating with AuNPs for DSA imaging to monitor the blood-vessel embolization. The temperature-sensitive nanogels enhanced the ability of DSA imaging because the gelation of nanogels dispersions could significantly reduce aggregation of AuNPs. AuNPs also sustained for long periods at the embolic sites which provided a long time to evaluate the efficacy of embolization therapy. Moreover, AuNPs increased the gelation strength of temperature-sensitive nanogels to improve the TAE therapeutic efficacy. In a rabbit renal artery model, gel dispersions had shown both effective blood vessel embolization and strong angiographic visibility, making them suitable for extended post-operative monitoring. Moreover, vascular recanalization was not observed for 28 days after the injection of the gel dispersion. The histological sections of kidney appeared coagulative necrosis, indicating this modified nanogels could effectively cut off the blood supply of the kidney over a long period [106].

**Fig. 4:**
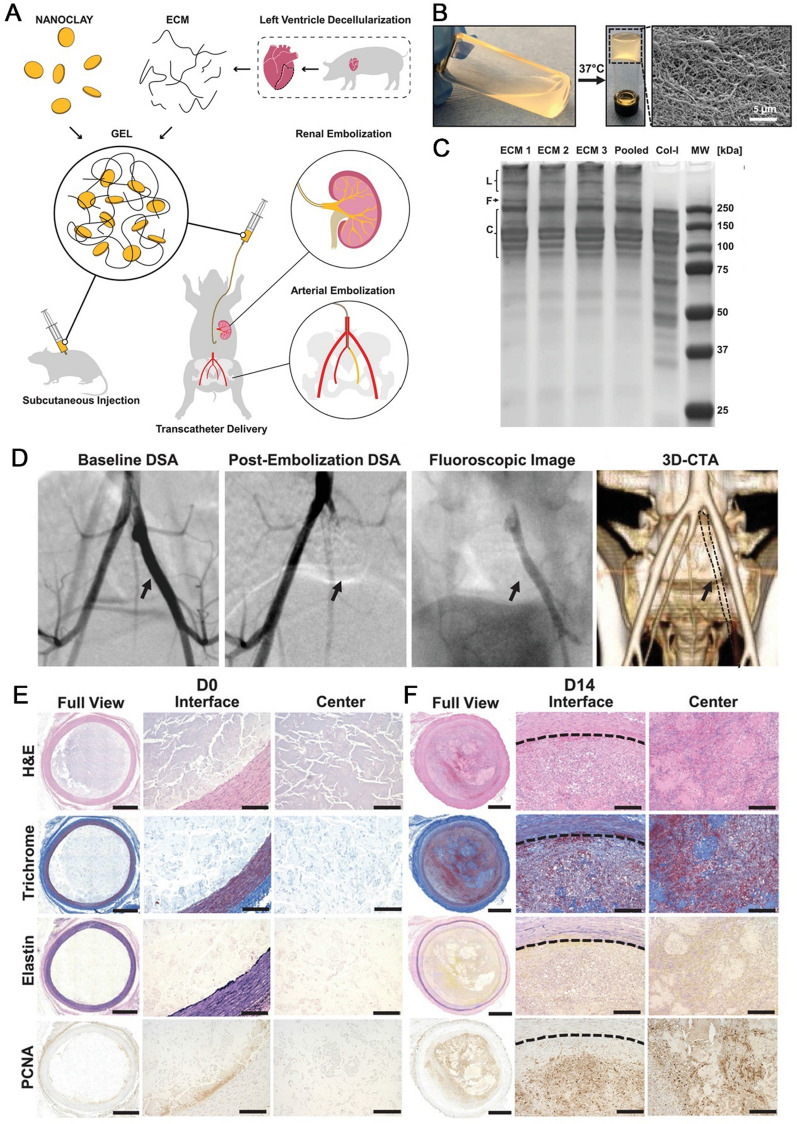
The ECM-derived biohybrid nanocomposites for permanent vessels embolization. **A** Schematics of ECM–NC nanocomposite gel fabrication. **B** ECM solution underwent sol–gel transition at 37 °C creating a nano-fibered mesh. **C** SDS–PAGE gel of ECM samples prepared from different porcine hearts. **D** Digitally subtracted angiography (DSA) of internal iliac artery under different condition. **E**, **F** Representative images of H&E, elastin, trichrome, and PCNA staining of embolized vessels at D0 (**E**), and D14 (**F**). Copyright 2020, Wiley

Recently, injectable decellularized extracellular matrix (ECM) hydrogels have emerged as a unique therapeutic platform for vessel embolization. Predominantly composed of polysaccharides such as glycosaminoglycans and hyaluronic acid, as well as fibrous proteins including collagen, elastin, fibronectin, and laminin. ECM provides a dynamic microenvironment. This ensures that ECM hydrogels maintain exceptional biocompatibility and biosafety [107, 108]. Study demonstrated that only porcine-derived cardiac ECM hydrogel obtained from the left ventricle of the heart has advanced into a clinical trial [109]. Besides, the cardiac ECM hydrogel can undergo sol–gel transition at body temperature, and this gelation effect may benefit embolotherapy by improving gel stability at targeted sites [110]. Nevertheless, the use of injectable cardiac ECM hydrogel in embolization therapy is hampered by its poor mechanical qualities and quick degradation profile. Hu et al. assembled the cardiac ECM hydrogel with Laponite nanoclay (NC) to obtain novel nanocomposite gels. ECM–NC nanocomposites were designed and fabricated by mixing 20 mg/mL ECM and 9 wt% Laponite NC (with an average hydrodynamic size of 7 nm) at predetermined ratios. This nanocomposite gels possessed shear-thinning characteristic allowing it to be injected from a wide range of micro and standard clinical catheters to achieve easy and rapid injection resulting in instant hemostasis. At body temperature, the ECM solution performed a sol–gel transition and exhibited enough mechanical strength. Importantly, the embolic area remained entirely occluded after two weeks indicating that mechanical stability of gels to obtain persistent occlusion without migration or fragmentation (Fig. 4) [111]. Above all, the innovative hybrid design that combines mechanical strength from synthetic nanoclay with tissue-based biological capabilities from ECM proteins signals a new approach in the endovascular therapy of vascular disorders.

Although there have been numerous attempts to overcome the drawbacks of already commercially available products, embolic agents that can fully meet the needs of clinics have not yet been invented. An ideal embolic agent requires the following aspects. Firstly, the agent should have an innate radiopacity to reduce various complications related to the additional mixing of other contrasting agents. Secondly, the system should have good biocompatibility to avoid neurotoxicity or vasospasm. Thirdly, agents should possess drug-loading capacity for releasing the therapeutics sustainably. Finally, the phase transition should be adjustable spatiotemporally, because it is vital to control the gelation penetration and rate depth through the body precisely to minimize the risks of catheter blockage and non-target embolization. Significantly, most studies do not keep track of the prognosis for long time. The long-term toxicity, stability, and biocompatibility of newly developed embolic agents are difficult to have an accurate assessment. More importantly, only a small number of embolic agents are moving forward to the clinical trial stage, and researchers also have not yet agreed on the standards for assessing embolic agents for clinical translation.

### Drug therapy

Vascular anomalies arise due to inherited or somatic genetic mutations. These mutations are predominantly found in genes that play essential roles in angiogenesis, lymphangiogenesis, vascular cell apoptosis, as well as growth and proliferation pathways. The major pathways include the PI3K (phosphoinositide 3-kinase)/AKT (protein kinase B)/mTOR (mammalian target of rapamycin), RAS (rat sarcoma)/RAF (rapidly accelerated fibrosarcoma)/MEK(mitogen-activated protein kinase kinase)/ERK (extracellular signal-regulated kinases), HGF (hepatocyte growth actor)/c-Met (hepatocyte growth factor receptor), and VEGF (vascular endothelial growth factor) A/VEGFR (vascular endothelial growth factor receptor) [112–114]. The exploiting targeted therapeutic agents largely focus on the above mutation-induced signaling.

Rapamycin, also known as sirolimus, is an inhibitor of the mTOR complex 1 (mTORC1), which can suppress downstream signaling and the associated protein synthesis, thereby reducing angiogenesis [115]. Propranolol, a first-line medicine for treating hemangiomas with great effectiveness, mainly involves the interaction between adrenergic and VEGF pathways [116]. Furthermore, the development of molecular agents targeting different mutations involved in angiogenesis and lymphangiogenesis is progressing. However, systemic administration methods, including oral and local injections, often lead to adverse effects, such as limited drug accumulation at the desired sites, low bioavailability, short half-life, off-target circulation, and toxicity to healthy tissues. Hence, there is a pressing need to explore effective and innovative strategies to enhance the pharmacological properties of drugs and improve the targeting capability and therapeutic efficacy in the treatment of vascular anomalies. In this section, we summarized the current development of biomaterials and nanotechnology of drug delivery systems with different methods of administration for vascular anomalies (Table 3).

**Table 3:** Summary of the development of nano-systems for the treatment of vascular anomalies

Nano-systems	Drug delivery	Treatment modalities	Surface modification	Application	Outcome	References
Liposomal gel	Propranolol hydrochloride	Transdermal drug delivery	–	Hemangiomas	Increase drug content in skin obviously, reduce dosing frequency compared with suspension, decrease drug content in the systemic circulation	[153]
Polymeric film	Propranolol hydrochloride	Transdermal drug delivery	–	Hemangiomas	Enhance skin retention, increases skin permeation ability	[126]
Cubic nanoparticles (CNPs)	Propranolol hydrochloride	Transdermal drug delivery	–	Hemangiomas	Improved drug transportation across, enhance skin retention with a decrease in the size of CNPs, possess favorable stability during storage	[127]
Lipid polymer nanoparticles	Rapamycin	Intravenous route	Anti-VEGFR2 antibody	Hemangiomas	Released rapamycin lastingly, and inhibiting hemangiomas both in vitro and in vivo	[154]
Poly(lactic-*co*-glycolic acid) (PLGA) nanoparticles	Propranolol	Intravenous route	Anti-VEGFR antibody	Hemangiomas	Anti-VEGFR antibody has a great targeted ability in HemECs and HUVECs	[25]
Polymer–lipid hybrid nanoparticles	Rapamycin	Intravenous route	–	Hemangiomas	Sustained release of rapamycin, and significantly reduced the frequency of administration	[155]
Exosome-mimetic nanoparticles-in-PLGA microspheres	Rapamycin	Intravenous route	–	Hemangiomas	Sustained release, efficient delivery, and therapeutic efficacy of rapamycin towards hemangiomas	[139]
Liposomes-in-microsphere (LIM)	Propranolol	Intravenous route	–	Hemangiomas	Promising approach to efficiently and locally deliver propranolol to the hemangioma, significant inhibit the infantile hemangioma	[156]
Poly(lactic-*co*-glycolic acid) (PLGA) nanoparticles	Propranolol	Intravenous route	CD133 aptamers	Hemangiomas	Significantly reduced the frequency of administration of propranolol and excellent targeted ability	[24]
Mesoporous silica nanoparticles(MSN)	Propranolol	Intravenous route	–	Hemangiomas	PRN@MSN inhibited the growth of hemangiomas both in vitro and in vivo and induced autophagy dysfunction with excessive autophagosome accumulation	[143]
Poly(ethylene oxide)–poly(d,l-lactic acid) (PEG-PLA)block copolymer	–	Intravenous route	Cell-penetrating peptide (CPP)	Vascular anomalies (human vascular networks)	Phototargeted NP can enhance accumulation of the NP and drug in a subcutaneous graft model of engineered vessels	[157]
Liposomes	Sodium morrhuate	Intravenous route	Anti-VEGFR2/KDR antibody	Hemangioma	KDR-targeting sodium morrhuate immunoliposomes have an increased capacity for binding to HECs and elicit apoptotic death through the mitochondrial apoptotic cascade	[158]
Gold nanoshells (AuNSs)	–	Intravenous route (photo-assist)	–	Venous malformation	Intravenously administered of AuNSs and then exposure by 808 nm NIR iodine laser can ablate venous malformations	[147]
Gold nanorods (GNRs)	–	Intravenous route (photo-assist)	CD31 antibody	Venous malformation	Anti-CD31 GNRs could be applied specifically to treat venous malformations and exhibited effective ablation of PTT both in vitro and in vivo	[148]

#### Transdermal drug delivery strategy

Transdermal drug delivery system (TDDS) refers to the route of drug delivery through the skin for local or systemic treatment which has proven to be one of the favorable methods among novel drug delivery systems. TDDS bypass the gastrointestinal tract, reducing the risk of liver metabolism. This not only minimizes the potential for gastrointestinal irritation but also decreases the chances of liver dysfunction, addressing the drawbacks associated with oral administration [117]. TDDS possesses a lot of advantages, including easy handling, minimum systemic exposure, controlled release, broad flexibility and tunability, and prolonged time of drug input. However, while the skin serves as a protective barrier shielding the body from pathogens, it also significantly impedes the transdermal penetration of drugs. The tough stratum corneum extremely limits the drugs across the skin. Therefore, enhancing skin permeability and achieving targeted delivery of therapeutic agents are essential challenges to enhance the treatment outcomes of vascular anomalies on the skin via transdermal drug delivery approaches [118].

**Fig. 5:**
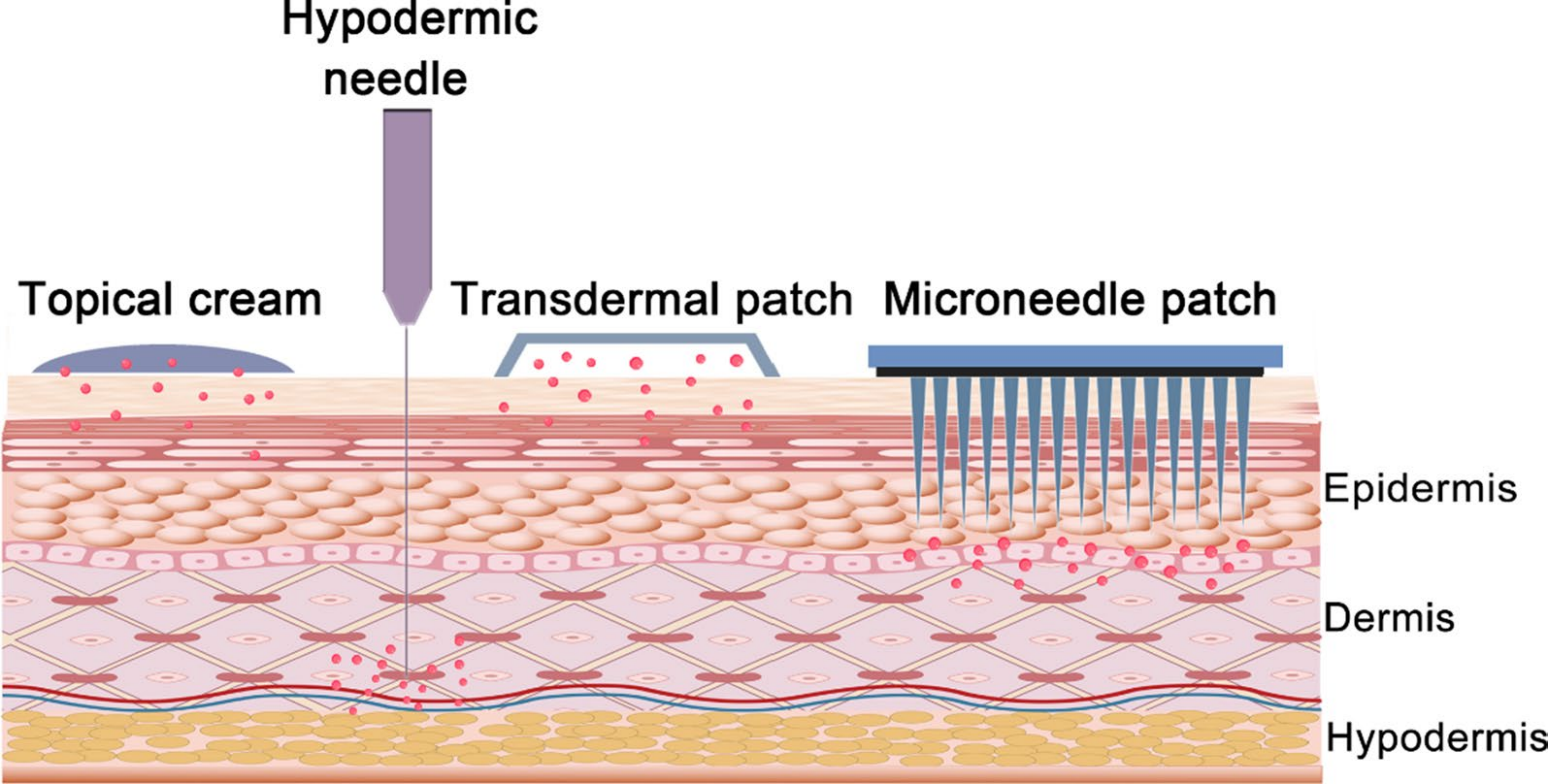
Comparison of topical cream, hypodermic needle, transdermal patch and microneedle patch

The traditional methods for transdermal drug delivery mainly include topical creams, transdermal patches, and hypodermic needles [119] (Fig. 5). Rapamycin and propranolol have been made into topical creams to treat superficial hemangioma and venolymphatic malformation effectively with no obvious side effects in the clinical observation [120–122]. However, the diffusion area is highly restricted. Studies have shown that the permeation of drugs loaded in cream formulations is typically confined to just 10–20% of the total drug content. This limitation might lead to incomplete fading of lesions, and some patients might not respond effectively to the superficial treatment [123]. Despite the ability of hypodermic needles to effectively deliver agents to subcutaneous lesions, their usage in therapy is restricted due to the need to puncture the epidermis, causing discomfort and pain for patients, particularly children. As a result, hypodermic needles are not commonly employed in treatment. Upon aforesaid cutaneous administration, in order to promote drug permeation ability through the skin, improve the bioavailability of agents and achieve precise delivery, innovative topical formulations including hydrogel, film, and liposomes have been developed [124–126]. Researchers have also investigated various nanocarrier-based drug transdermal delivery systems. Based on their chemical and physical properties, nanocarriers have the capability to penetrate various distinct layers of the skin structure, offering potential benefits in targeted drug delivery and enhancing the efficacy of dermatological treatments. Zeng et al. reported cubic nanoparticles (CNPs) could enhance transdermal permeation and had the potential to treat hemangioma in the deep of the skin. The cubic NPs, also known as liquid crystalline nanoparticles, were usually composed of amphiphilic lipids, phytantriol, and stabilizers to form cubic structures for the delivery of small bio-macromolecules and molecules. The CNPs not also disrupted the structure of the lipid bilayer in the stratum corneum to enhance the fluidity of the stratum corneum and promote transdermal penetration, but also had a similar cubic structure to the cell membranes. The skin retention of the PRN@CNPs was also enhanced with a decrease in the size of CNPs. The CNPs with stable size distribution and high encapsulation efficiency significantly improved drug permeation ability across the skin both in vitro and in vivo [127].

Over the past few years, microneedles (MNs) have garnered significant interest as a promising approach in the treatment of various skin vascular anomalies. This technique can overcome the disadvantages associated with conventional transdermal drug delivery methods [128]. Microneedles, composed of hundreds of micro-scaled needles arranged in an array, enable penetration through the skin barrier. This design facilitates the simultaneous delivery of various therapeutic compounds, including small hydrophilic molecules, peptides, proteins, DNA, and NPs [129]. The refined MNs structure can break the stratum corneum layer for a clear pathway allowing more therapeutic drugs to penetrate skin and cause minimal invasiveness to patients. Various types of MNs have been developed and explored for drug delivery, predominantly including solid, coated, hollow, and dissolving MNs. Each kind of MNs has a unique method for delivering agents to the epidermis. Some are only employed to make pores in the stratum corneum, some have the drug solution precoated on their surface, some are dissolvable, and some have the drug solution prefilled inside of them [130]. However, the solid, coated, hollow type of MNs are usually made of rigid structural materials such as silicon, metals, or silica glass. The tough needle structure may result in severe damage to the skin when needles fracture and are left in the skin.

Dissolving MNs are crafted from biodegradable polymers, including hyaluronic acid (HA), polyvinyl pyrrolidone (PVP), maltose, and albumin. Drugs can simultaneously encapsulate within these polymers. Upon insertion into the skin, the microneedle structure dissolves and degrades, releasing the drugs at the mean time. This design substantially minimizes the risk of injuries from residual needle fragments [131]. Because of the bio-acceptability and dissolution of polymer inside the skin, dissolving MNs is one of the finest options for long-term therapy with enhanced patient compliance. Efficient needle drug distribution is a significant issue that must be addressed while designing dissolving MNs. Hence, polymer-drug mixing is a critical step in such fabrication. He et al. developed a dissolving MNs encapsulating propranolol hydrochloride via a two-step casting procedure. Hyaluronic acid (HA) and polyvinyl pyrrolidone (PVP)-K90 were merged as the matrix materials of the needles. Prepared only by HA had high fragility, whereas PVP-K90 was a polymer with good toughness and hardness. The needles were arranged in a 12 × 12 array in a patch of 8 mm × 8 mm with a center-to center interval of 600 µm. These MNs possessed enough strength to pierce stratum corneum, and enhanced skin retention and cumulative permeation of propranolol hydrochloride. Meanwhile, HA-mediated skin hydration could serve as a drug reservoir, allowing sustained drug release [132]. Mao et al. synthesized a relative polyvinyl alcohol (PVA) matrix MNs encapsulating with rapamycin (RAPA) to treat vascular anomalies by anti-angiogenesis mechanism. The fabricated RAPA MNs had a structure of pyramidal shape containing 100 needles with an average length of 800 mm, and had excellent dissolving ability. Additionally, the PVA matrix enhanced the water-dissolvability of rapamycin, which could be released from the microneedles swiftly and efficiently. The MNs exhibited efficient anti-angiogenesis ability and had good practical safety both in vitro and in vivo [133]*.*

While transdermal drug delivery systems (TDDS) show promise as a treatment option for different vascular skin lesions, they face certain challenges that need to be addressed. These challenges include the intricate application process when combined with other drug delivery formulations, the duration of sustained controlled release of drugs, and the safety concerns associated with the clinical application of MNs. Overcoming these challenges will pave the way for the successful implementation of TDDS and provide effective treatment options for patients with vascular skin lesions.

#### Intravenously injected nano-drug delivery systems

For the delivery of therapeutic medicines to various vascular associated disorders, a wide variety of targeted ligands as surface modification of NPs are now being explored intensively. Targeting vascular endothelial growth factor receptor (VEGFR) can inhibit the proliferation and migration of endothelial cells. Hence, VEGFR plays an important role in the application of both lymphatic and vascular diseases. VEGFR consists of three tyrosine kinase receptors including VEGFR-1, VEGFR-2, and VEGFR-3. VEGFR-2 is the essential receptor for the formation of the blood vasculature, whereas lymphogenesis mostly involves VEGFR-3 [134–136]. Thus, VEGFR is a valuable target aiming to vascular anomalies for the surface modification of NPs and widely applied in the few decades.

**Fig. 6:**
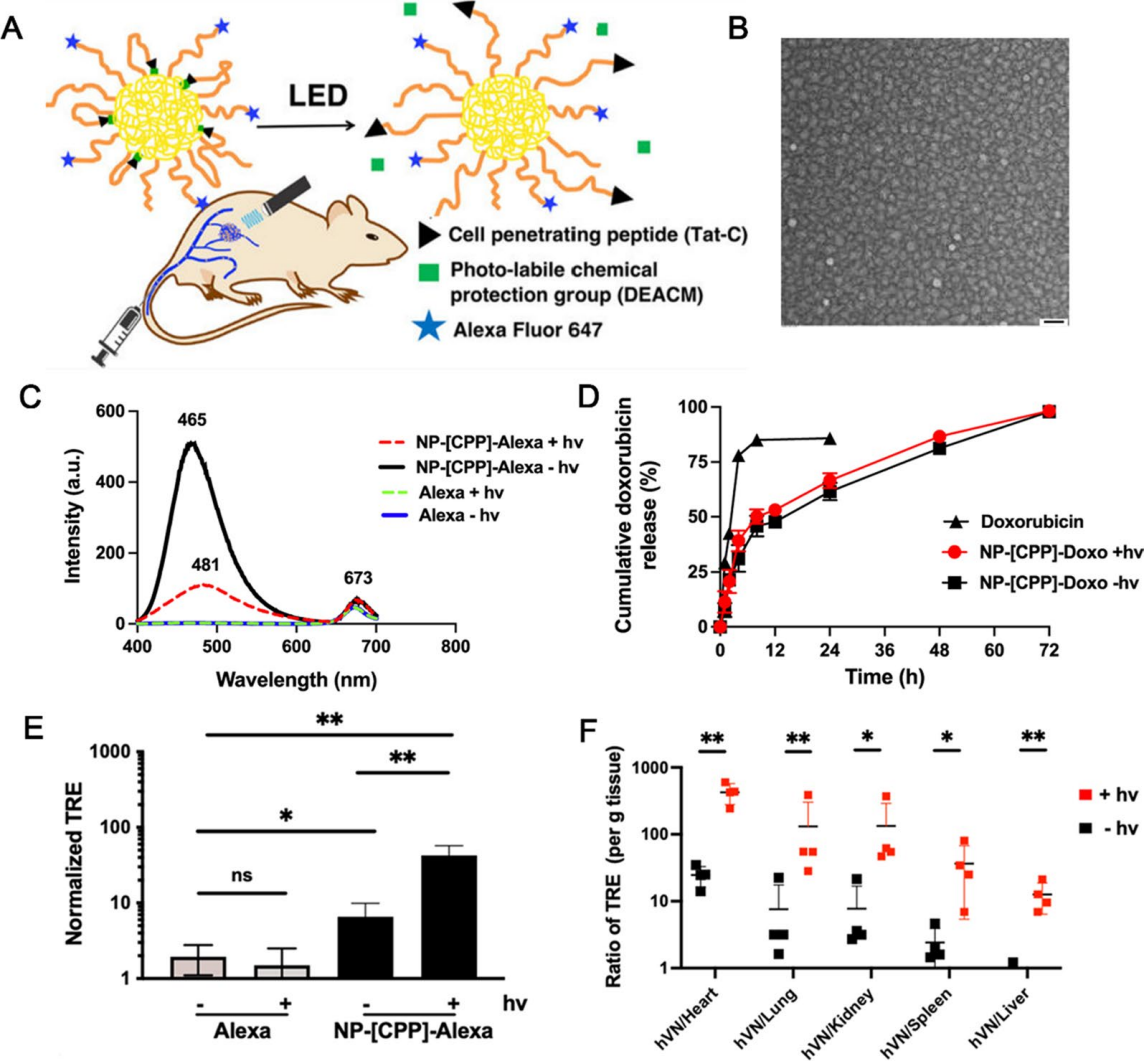
Targeting nanoparticles to bioengineered human vascular networks. **A** Schematic of light-triggered nanoparticles. **B** TEM image of NP-[CPP]-Alexa. Scale bar: 50 nm; **C** Fluorescence emission spectrum of NP-[CPP]-Alexa or Alexa-647 fluorophore in PBS with and without irradiation (hν) for 1 min. **D** Cumulative doxorubicin release from nanoparticles with or without irradiation. **E** Phototargeting of nanoparticles in vivo in the hVN model. Total radiant efficiency (TRE) in hVN 24 h after IV injection. **F** Ratio of total radiant efficiency (TRE, per gram of tissue) in the hVNs to TRE (per gram of tissue) in various other organs, in the presence (red) and absence (black) of irradiation. Copyright 2021, American Chemical Society

Moreover, the emerging targeting molecules haves been designed currently. Cullion et al. used cell-penetrating peptide (CPP) as the photo-triggered targeting modification to target human vascular networks (hVNs) aiming at the treatment of vascular anomalies [137]. Photo-targeted NPs were formed by self-assembly of a poly (ethylene oxide)–poly (d,l-lactic acid) (PEG–PLA) block copolymer and then modified with a Tat-C cell-penetrating peptide as the targeting moiety to promote cellular accumulation. With photo-targeting modification, there was 10 folds more NPs accumulation within hVNs model than in other major organs (Fig. 6). Exosomes have been shown to inherently target specific cells and evade rapid clearance by the immune system, because of the unique surface molecules [138]. Li et al. developed exosome-mimetic NPs encapsulating rapamycin (RN) and subsequently incorporated these into microspheres (RNM). The design of RNM aimed to prolong the release duration of rapamycin, due to the sustained release properties of both NPs and microspheres. Notably, the RN released from PLGA microspheres retained its targeting capability and exhibited specific inhibitory effect against hemangiomas [139]. Besides, researchers have assembled numerous nano-drug delivery systems with specific surface modification to achieve better therapeutic effects. We summarized the different nano-systems in the therapy of vascular anomalies (Table 3).

**Fig. 7:**
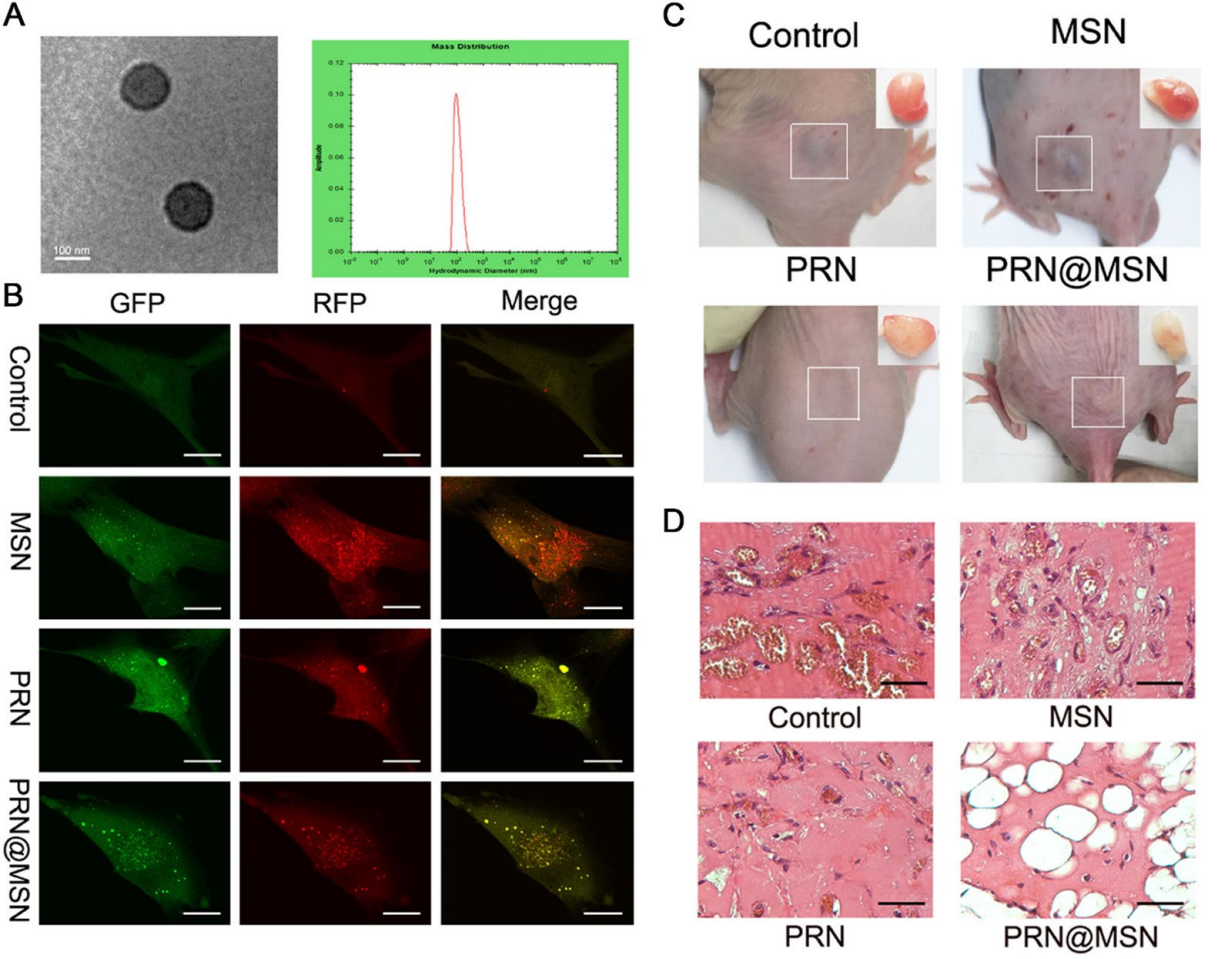
**A** The characterization of PRN@MSN. **B** The change of autophagic flux detected by confocal microscopy in HemSCs transfected with mRFP-GFP-LC3 adenovirus after treated with PRN@MSN. **C** The antitumor effect of PRN@MSN in vivo. D The H&E staining of animal modes with hemangiona. Copyright 2020, Elsevier

Beyond advent of nanotechnology in drug delivery, NPs are increasingly recognized for potential in interacting with the cellular machinery that further govern cell fate and activity. NPs hold the ability to generate reactive oxygen species (ROS) or initiate signaling pathways that can not only regulate but also eventually alter various cellular responses, including necrosis, necroptosis, apoptosis, autophagy, and mitotic catastrophe. The specific cell response to NPs is complex and determined by many diverse factors, mainly including surface charge, crystallinity, ligand specificity, and surface chemistry. Consequently, research is underway to determine properties and functional groups that can have an influence on cellular outcomes and biological responses of NPs. Just as mesoporous silica nanoparticles (MSN) could modulate autophagic activities through various signaling pathways in different cells [140, 141]. However, autophagy, serving as a cellular protection and survival mechanism, may contribute to tumor development and progression in the later stages [142]. Hence, while the intended purpose of MSN is drugs delivery to the target cells, the activation of autophagy could promote cell survival, potentially mitigating the therapeutic efficacy of the anti-tumor drugs. In view of this, our group encapsulated the propranolol into MSN in the therapy of infantile hemangioma (IH). Propranolol has the cytotoxic, antiproliferative, and antiangiogenic effects to induce apoptosis. More importantly, PRN has been reported that can regulate the late autophagic process via blocking the autophagosome and lysosome. PRN@MSN induced autophagosome formation in the assistance of MSN and blocked the degradation of autophagosomes induced by PRN, leading to excessive autophagosome accumulation which directly resulted in the cell death. PRN and MSN had a synergistic effect, on the one hand, the application of PRN changed the pro-survival stage of autophagy occurred by MSN; on the other hand, the application of MSN was an ideal carrier to assist more therapeutic agents accumulation in the lesion area, result in the strongest inhibitory effect in vitro and vivo (Fig. 7) [143].

Above all, identifying or distinguishing the specific cellular and extracellular biomarkers of different vascular diseases is critical for the design and synthesis of NPs. Hence, the commonly specific cellular and extracellular markers in vascular anomalies were summarized, such as Glucose transporter 1 (Glut-1) expressed specifically in infantile hemangioma. Such common biomarkers could assist researchers to design and develop the targeting NPs (Table 4). Moreover, future studies should focus more on obtaining a detailed understanding of how NPs interact with cells or therapeutic agents at molecular and functional levels in order to design the most appropriate nano drug delivery system while maximizing the therapeutic effects.

**Table 4:** The potential biomarker in design of targeted nanomaterials in vascular anomalies

Cellular and extracellular markers	
Vascular tumors	
Hemangioma Kaposiform hemangioendotheliomas Angiosarcoma	Glucose transporter 1 (Glut-1), Apelin receptor (APJ), CD31, CD34, CD133, FCg II receptor (FcgRII) [7, 8] PROX-1, D2-40, Claudin-5, VEGFR3, CD34, CD31, Fli-1, ERG, Claudin-5, D2-40 [11] CD31, CD34, Prox-1, Fli-1 [14]
Vascular malformations	
Capillary malformations Lymphatic malformations Venous malformations Arteriovenous malformations	CD133, CD166, EphB1, EfnB2 [159] D2-40, LYVE-1, VEGFR-3 [160] CD31 [148] –

### Photo-assisted therapy

Photo-assisted therapy has undergone extensive development as a potent method for light-activated cytotoxicity and non-invasive treatment. This method achieves targeting accuracy through the binding of specific markers on lesion regions from the delivered therapeutic agents, combined with precise light irradiation directed at the affected areas. Until now, photothermal treatment (PTT) and photodynamic therapy (PDT) have been two photo-assisted therapeutic procedures that have been most frequently investigated with potential in vascular diseases. The laser therapy obtains great therapeutic effects in vascular lesions with less adverse events, specifically in hemangioma with at least a superficial component and capillary malformations [144].

PTT regularly uses photothermal conversion agents (PTAs) to produce enough heat during near-infrared (NIR) light irradiation to induce cell death (temperatures higher than 45 °C) or enhance the sensitivity of cells to other therapy (temperatures between 40 and 45 °C). Without laser irradiation, PTAs are relatively safe for cells and almost nontoxic [145]. PTT possesses some attractive advantages, such as reduced side effects, minimal invasiveness, and high specificity. Moreover, PTT has a great selectivity due to the controllability of various laser irradiation parameters (e.g., laser wavelength, location, light intensity and irradiation time). In the previous therapy, outside-in methods were frequently used to raise the temperature of target tissue. Techniques such as regional hyperthermia, superficial hyperthermia, radiofrequency, and microwaves were used. These methods create a temperature gradient that peaks at the surface of body and diminishes as it moves away from the external heat source. This can inadvertently affect healthy tissue, leading to potential side effects. Therefore, exploring photothermal agents (PTAs), which are pivotal components of PTT, is crucial for inducing a localized temperature rise. To overcome the limitations of traditional hyperthermia techniques, NPs arewere used to produce heat in response to external stimuli. NPs accumulate in the lesion tissue and irradiate with the NIR laser, then NPs active in specific areas and convert the NIR laser energy into heat and finally induce a localized hyperthermia effect. The local irradiation can minimize damage to surrounding healthy tissue to the largest extent. A primary focus for researchers has been the light-to-heat conversion efficiency of these NPs. To ensure the most efficient light/heat conversion in PTT, it is necessary for NPs to have high absorption efficiency and low luminescence capacity. Among the inorganic nanomaterials explored for mediating a tumor-localized photothermal effect mainly include gold, carbon-based materials, tungsten, copper, molybdenum, and iron oxide [146].

**Fig. 8:**
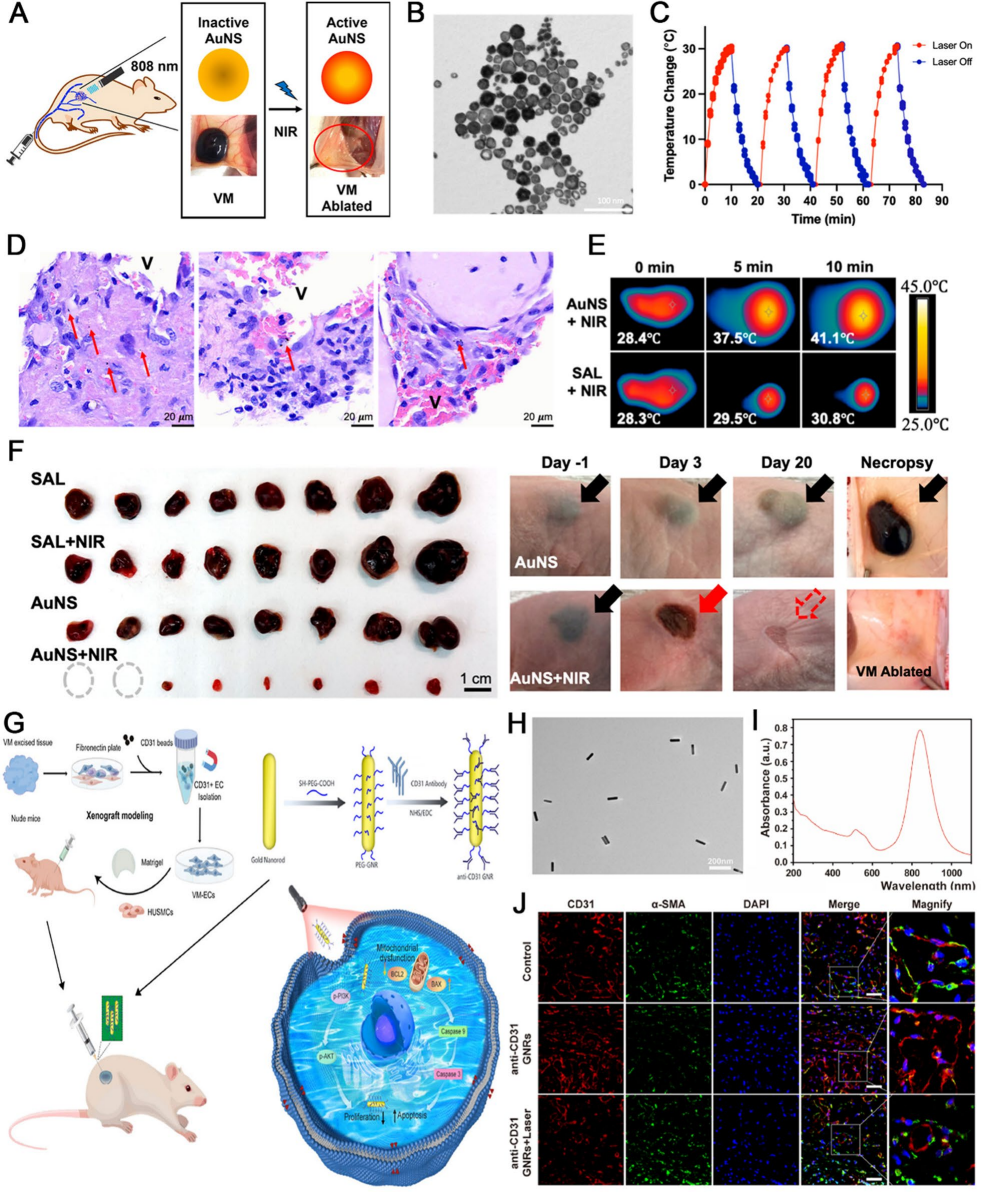
The photothermal therapy in vascular anomalies. **A** The synthesis strategy. **B** TEM image of gold nanoshells (AuNSs). **C** Photothermal characteristic. **D** H&E staining shown AuNS aggregated accumulation (red arrow pointing to dark spots) 24 h after tail vein injection. **E** Thermal images of VMs followed by NIR irradiation in vivo. **F** VM explants after different treatments. Copyright 2023, American Chemical Society. **G** Schematic design of CD31 antibodies conjugated with PEG-GNRs. **H** TEM image of GNRs. **I** UV–vis spectrum of the GNRs' solution; **J** Treatment effect of NIR irradiation. Immunofluorescence staining of CD31, α-SMA, and DAPI. Copyright 2022, Elsevier

Recently, researchers have started to explore PTT therapy in the vascular system. The primary focus of these studies has been on gold element, due to its unique properties. Gold nanoshells (AuNSs) had been shown to accumulate within vascular malformations (VMs) following intravenous injection. Upon irradiation, AuNSs could induce a potent photothermal effect, effectively eliminating VMs. The AuNSs had spherical morphology under the observation of transmission electron microscopy (TEM). Upon the irradiation of 808 nm NIR laser, the AuNSs had a rapid temperature increase and could be triggered repeatedly. Additionally, the photothermal therapy significantly reduced or even eliminated VMs, exhibiting excellent therapeutic effect (Fig. 8A–F) [147]. To further enhance the accumulation of NPs in the targeted site, Jiang et. al. investigated that gold nanorods (GNRs) combined with cell adhesion molecule-1 (CD31) antibody. The CD31 is a marker for endothelial cells which is highly expressed in the membrane of venous malformation endothelial cells (VM-ECs). The synthesized GNRs were first modified with polyethylene glycol (PEG) to avoid nanoparticle agglomeration, and then CD31 antibody were conjugated to the surface of the GNRs via electrostatic adherence. The anti-CD31 GNRs with non-toxic and good biocompatible properties exhibited a synergistic biological effect by promoting apoptosis and inhibiting proliferation on VM-ECs and significantly reduced the lumen area of VM xenograft model followed by 808 nm NIR laser [148] (Fig. 8G–J). Above all, near-Infrared (NIR) light, which allows for deep tissue penetration, could be suitable for treating VMs. However, VMs can be extensive, spanning tens of centimeters. In such instances, it may be necessary to administer NPs or perform irradiation repeatedly. Even when irradiation can only ablate a portion of a VMs, this approach could aid surgical resection. The removal of scar tissue might pose a lower risk of severe hemorrhage compared to the resection of a large, vascularized VMs.

PDT primarily utilizes the generation of reactive oxygen species (ROS) to induce cytotoxic effects. This is achieved by activating a photosensitizer (PS) with visible or NIR light, typically requiring a lower-power modality compared to PTT. The light, PS, and oxygen are essential for PDT. Photosensitizers become core of the PDT system when exposed to specific light wavelengths. Therefore, the overall light-conversion efficiency of the entire PDT system mainly depends upon the photosensitizers [149]. The light-induced products of photosensitizer also provide the origin for the PDT to exhibit therapeutic effects. Despite the fact that there are many different forms of photosensitizers, researchers have revealed that two main types of photosensitizers typically have excellent therapeutic benefits, as follows: (1) Porphyrin and its derivatives, including porphyrin precursors, (2) inorganic nanoplatforms. Hematoporphyrin derivative (HpD) as the first-generation photosensitizers have been wildly used in the therapy of vascular malformations, especially in capillary malformation [150]. However, there were two significant issues. Light cannot penetrate deeply into tissue because of the short absorption peak wavelength. Meanwhile, generalized photosensitivity requires photoprotection for a few weeks depending on the long half-life of photosensitizers [151]. In order to solve such limitations and increase the accumulation of the agent in the vascular lesion, Orbay et al. developed a porphyrin-based nano-compounds which possesses a smaller size (25 nm), leading to selectively accumulate in the animal mode of IH. Based on this nanoplatforms, the animal models of hemangiomas began regression within just one day following treatment. Furthermore, the hemangiomas completely disappeared after 21 days therapy [152]. Significantly, the development of various NPs and the exploration of related therapeutic strategies provide powerful tools to overcome the challenges faced by photosensitizers, serving either as carriers or as photosensitizers themselves. However, research specifically targeting nanocarrier drug delivery systems designed for vascular diseases remains insufficient. Given the potential of PDT in revolutionizing therapeutic approaches, there is an urgent need to delve deeper into this area via the nanoplatforms.

## Conclusion and outlook

Congenital vascular anomalies are recognized to be the most complicated vascular disease and can be classified into two types: vascular tumors and vascular malformations. Various types of nanoscale agents or biomaterials have been investigated, allowing researchers to overcome limitations of conventional diagnosis and therapy methods. The use of nanotechnology can observe clearer vascular or lymphatic structures imaging and has the ability to distinguish the different vascular diseases, allowing for early detection and assist to the early therapy. The multifunctional nature of NPs also allows for multimodal imaging, merging different imaging modalities to enhance diagnostic accuracy. In therapy, nanotechnology can achieve high delivery efficacy of therapeutic agents while minimizing systemic side effects. Meanwhile, the nano-systems can improve the traditional therapeutic materials to achieve precise treatment and reduce invasiveness.

However, the nanotechnology applying in vascular anomalies are still in the beginning, vascular anomalies should receive more attention by researchers. Looking ahead, there are several areas that warrant further exploration and development in the diagnosis and therapy of vascular anomalies. In diagnosis, the synthesis process of various nano contrast agents should be easier to achieve quantitative production as much as possible. Manufacturing processes for nanomaterials must also be reproducible to ensure consistent quality and performance of the imaging agents. In addition, the imaging characteristics of NPs are significantly influenced by various factors including size, surface properties, morphology, and materials; hence, a thoughtful design that comprehensive consideration of both biological and physicochemical properties is essential for preparing effective nano contrast agents or nanoprobes. Besides, the interactions between different imaging modalities and materials are currently not well understood and lack systematic study. Further exploration of the underlying mechanisms and subsequent bioeffects at the cellular, molecular, and organic levels is required before clinical application. More importantly, obtaining approval from regulatory agencies represents a substantial obstacle in the clinical translation of nano contrast agents. Thus, to facilitate more effective clinical applications, the biocompatibility, metabolic pathways and long-term toxicity of various contrast agents in body is a top priority for our future exploration and research. As for the therapeutic aspects of vascular anomalies, firstly, aiming at stubborn vascular anomalies or lesion at important site, developing the novel molecular targeted drugs and drugs delivery method is urgent. Moreover, more research is needed to address the challenges associated with the translation of nanotechnology into clinical practice. Issues such as scalability, reproducibility, and safety considerations need to be thoroughly investigated and addressed. Additionally, the long-term effects and potential toxicity of NPs or biomaterials in the human body need to be carefully evaluated.

In summary, the recent advances in nanotechnology have shown great promise in revolutionizing the diagnosis and therapy of vascular anomalies. As further research is conducted and technological advancements continue, we can expect significant improvements in early detection, precise targeting, and effective treatment strategies for vascular anomalies, ultimately leading to better outcomes for patients suffering from these conditions.

## Data Availability

No datasets were generated or analysed during the current study.

## References

[1] Wassef M, Blei F, Adams D, Alomari A, Baselga E, Berenstein A, Burrows P, Frieden IJ, Garzon MC, Lopez-Gutierrez JC (2015). Vascular anomalies classification: recommendations from the international society for the study of vascular anomalies. Pediatrics.

[2] Kunimoto K, Yamamoto Y, Jinnin M (2022). Issva classification of vascular anomalies and molecular biology. Int J Mol Sci.

[3] Bertino F, Trofimova AV, Gilyard SN, Hawkins CM (2021). Vascular anomalies of the head and neck: diagnosis and treatment. Pediatr Radiol.

[4] Flores AM, Ye J, Jarr K, Hosseini-Nassab N, Smith BR, Leeper NJ (2019). Nanoparticle therapy for vascular diseases. Arterioscler Thromb Vasc Biol.

[5] Cohen MJ (2006). Vascular update: morphogenesis, tumors, malformations, and molecular dimensions. Am J Med Genet A.

[6] Jung HL (2021). Update on infantile hemangioma. Clin Exp Pediatr.

[7] Sun Y, Qiu F, Hu C, Guo Y, Lei S (2022). Hemangioma endothelial cells and hemangioma stem cells in infantile hemangioma. Ann Plast Surg.

[8] North PE, Waner M, Mizeracki A, Mihm MJ (2000). Glut1: a newly discovered immunohistochemical marker for juvenile hemangiomas. Hum Pathol.

[9] Ji Y, Chen S, Yang K, Xia C, Li L (2020). Kaposiform hemangioendothelioma: current knowledge and future perspectives. Orphanet J Rare Dis.

[10] Adams DM, Hammill A (2014). Other vascular tumors. Semin Pediatr Surg.

[11] Le Huu AR, Jokinen CH, Rubin BP, Mihm MC, Weiss SW, North PE, Dadras SS (2010). Expression of prox1, lymphatic endothelial nuclear transcription factor, in kaposiform hemangioendothelioma and tufted angioma. Am J Surg Pathol.

[12] Florou V, Wilky BA (2021). Current management of angiosarcoma: recent advances and lessons from the past. Curr Treat Options Oncol.

[13] Rosenberg A, Agulnik M (2018). Epithelioid hemangioendothelioma: update on diagnosis and treatment. Curr Treat Options Oncol.

[14] Goerdt LV, Schneider SW, Booken N (2022). Cutaneous angiosarcomas: molecular pathogenesis guides novel therapeutic approaches. J Dtsch Dermatol Ges.

[15] Huang JT, Liang MG (2010). Vascular malformations. Pediatr Clin North Am.

[16] Escobar K, Pandher K, Jahnke MN (2022). Capillary malformations. Dermatol Clin.

[17] Elluru RG, Balakrishnan K, Padua HM (2014). Lymphatic malformations: diagnosis and management. Semin Pediatr Surg.

[18] Kulungowski AM, Patel M (2020). Lymphatic malformations. Semin Pediatr Surg.

[19] Cooke-Barber J, Kreimer S, Patel M, Dasgupta R, Jeng M (2020). Venous malformations. Semin Pediatr Surg.

[20] Schimmel K, Ali MK, Tan SY, Teng J, Do HM, Steinberg GK, Stevenson DA, Spiekerkoetter E (2021). Arteriovenous malformations-current understanding of the pathogenesis with implications for treatment. Int J Mol Sci..

[21] Arneja JS, Gosain AK (2008). Vascular malformations. Plast Reconstr Surg.

[22] Cheng Z, Li M, Dey R, Chen Y (2021). Nanomaterials for cancer therapy: current progress and perspectives. J Hematol Oncol..

[23] Dhaliwal A, Zheng G (2019). Improving accessibility of epr-insensitive tumor phenotypes using epr-adaptive strategies: designing a new perspective in nanomedicine delivery. Theranostics.

[24] Guo X, Zhu X, Gao J, Liu D, Dong C, Jin X (2017). Plga nanoparticles with cd133 aptamers for targeted delivery and sustained release of propranolol to hemangioma. Nanomedicine (Lond).

[25] Zhu X, Guo X, Liu D, Gong Y, Sun J, Dong C (2017). Promotion of propranolol delivery to hemangiomas by using anti-vegfr antibody-conjugated poly(lactic-*co*-glycolic acid) nanoparticles. J Biomed Nanotechnol.

[26] Schiffelers RM, Koning GA, Ten HT, Fens MH, Schraa AJ, Janssen AP, Kok RJ, Molema G, Storm G (2003). Anti-tumor efficacy of tumor vasculature-targeted liposomal doxorubicin. J Control Release.

[27] Swain S, Sahu PK, Beg S, Babu SM (2016). Nanoparticles for cancer targeting: current and future directions. Curr Drug Deliv.

[28] Kumari P, Ghosh B, Biswas S (2016). Nanocarriers for cancer-targeted drug delivery. J Drug Target.

[29] Das A, Goyal A, Sangwan A, Bhalla AS, Kumar A, Kandasamy D, Dawar R (2023). Vascular anomalies: nomenclature, classification, and imaging algorithms. Acta Radiol.

[30] Holowka S, Shroff M, Chavhan GB (2019). Use and safety of gadolinium based contrast agents in pediatric mr imaging. Indian J Pediatr.

[31] Boretius S, Frahm J (2011). Manganese-enhanced magnetic resonance imaging. Methods Mol Biol.

[32] Longmire MR, Ogawa M, Choyke PL, Kobayashi H (2014). Dendrimers as high relaxivity mr contrast agents. Wiley Interdiscip Rev Nanomed Nanobiotechnol.

[33] Kobayashi H, Kawamoto S, Jo SK, Bryant HJ, Brechbiel MW, Star RA (2003). Macromolecular MRI contrast agents with small dendrimers: pharmacokinetic differences between sizes and cores. Bioconjug Chem.

[34] Lim J, Turkbey B, Bernardo M, Bryant LJ, Garzoni M, Pavan GM, Nakajima T, Choyke PL, Simanek EE, Kobayashi H (2012). Gadolinium mri contrast agents based on triazine dendrimers: relaxivity and in vivo pharmacokinetics. Bioconjug Chem.

[35] Polasek M, Hermann P, Peters JA, Geraldes CF, Lukes I (2009). Pamam dendrimers conjugated with an uncharged gadolinium(iii) chelate with a fast water exchange: the influence of chelate charge on rotational dynamics. Bioconjug Chem.

[36] Fu S, Cai Z, Liu L, Fu X, Wu C, Du L, Xia C, Lui S, Gong Q, Song B (2023). Gadolinium(iii) complex-backboned branched polymers as imaging probes for contrast-enhanced magnetic resonance angiography. ACS Appl Mater Interfaces.

[37] Geninatti CS, Bussolati B, Tei L, Grange C, Esposito G, Lanzardo S, Camussi G, Aime S (2006). Magnetic resonance visualization of tumor angiogenesis by targeting neural cell adhesion molecules with the highly sensitive gadolinium-loaded apoferritin probe. Cancer Res.

[38] Dai H, Shen Q, Shao J, Wang W, Gao F, Dong X (2021). Small molecular nir-ii fluorophores for cancer phototheranostics. Innovation (Camb).

[39] Liu Z, Zhao M, Wang H, Fu Z, Gao H, Peng W, Ni D, Tang W, Gu Y (2022). High relaxivity gd(3+)-based organic nanoparticles for efficient magnetic resonance angiography. J Nanobiotechnol.

[40] He F, Zhu L, Zhou X, Zhang P, Cheng J, Qiao Y, Feng Y, Yue S, Xu M, Guan J (2022). Red blood cell membrane-coated ultrasmall nagdf4 nanoprobes for high-resolution 3d magnetic resonance angiography. Acs Appl Mater Interfaces.

[41] Yano K, Matsumoto T, Okamoto Y, Kurokawa N, Hasebe T, Hotta A (2021). Fabrication of GD-dota-functionalized carboxylated nanodiamonds for selective MR imaging (MRI) of the lymphatic system. Nanotechnology.

[42] Sohn CH, Park SP, Choi SH, Park SH, Kim S, Xu L, Kim SH, Hur JA, Choi J, Choi TH (2015). MRI molecular imaging using glut1 antibody-Fe_3_O_4_ nanoparticles in the hemangioma animal model for differentiating infantile hemangioma from vascular malformation. Nanomedicine (Lond).

[43] Guneyli S, Ceylan N, Bayraktaroglu S, Acar T, Savas R (2014). Imaging findings of vascular lesions in the head and neck. Diagn Interv Radiol.

[44] Annapragada AV, Hoffman E, Divekar A, Karathanasis E, Ghaghada KB (2012). High-resolution CT vascular imaging using blood pool contrast agents. Methodist Debakey Cardiovasc J.

[45] de Vries A, Custers E, Lub J, van den Bosch S, Nicolay K, Grull H (2010). Block-copolymer-stabilized iodinated emulsions for use as CT contrast agents. Biomaterials.

[46] Attia MF, Anton N, Akasov R, Chiper M, Markvicheva E, Vandamme TF (2016). Biodistribution and toxicity of x-ray iodinated contrast agent in nano-emulsions in function of their size. Pharm Res.

[47] Dekrafft KE, Xie Z, Cao G, Tran S, Ma L, Zhou OZ, Lin W (2009). Iodinated nanoscale coordination polymers as potential contrast agents for computed tomography. Angew Chem Int Ed Engl.

[48] Zou Y, Wei Y, Wang G, Meng F, Gao M, Storm G, Zhong Z (2017). Nanopolymersomes with an ultrahigh iodine content for high-performance x-ray computed tomography imaging in vivo. Adv Mater.

[49] Yu Y, Yang T, Sun T (2020). New insights into the synthesis, toxicity and applications of gold nanoparticles in ct imaging and treatment of cancer. Nanomedicine (Lond).

[50] Silva F, Cabral CM, Paulo A (2020). Radiolabeled gold nanoparticles for imaging and therapy of cancer. Materials (Basel)..

[51] Hainfeld JF, Ridwan SM, Stanishevskiy Y, Smilowitz NR, Davis J, Smilowitz HM (2018). Small, long blood half-life iodine nanoparticle for vascular and tumor imaging. Sci Rep..

[52] Khademi S, Sarkar S, Shakeri-Zadeh A, Attaran N, Kharrazi S, Ay MR, Azimian H, Ghadiri H (2019). Targeted gold nanoparticles enable molecular CT imaging of head and neck cancer: an in vivo study. Int J Biochem Cell Biol.

[53] Fernandez IS, Roldan FA, Merino LT, Fernandez RS, Mera CM, Gullon GR (2019). Ultrasound elastography images in vascular anomalies. J Ultrasound Med.

[54] Wang S, Hossack JA, Klibanov AL (2018). Targeting of microbubbles: contrast agents for ultrasound molecular imaging. J Drug Target.

[55] Guvener N, Appold L, de Lorenzi F, Golombek SK, Rizzo LY, Lammers T, Kiessling F (2017). Recent advances in ultrasound-based diagnosis and therapy with micro- and nanometer-sized formulations. Methods.

[56] Perera RH, Hernandez C, Zhou H, Kota P, Burke A, Exner AA (2015). Ultrasound imaging beyond the vasculature with new generation contrast agents. Wiley Interdiscip Rev Nanomed Nanobiotechnol.

[57] Yu H, Li C, He X, Zhou Q, Ding M, Duric N, Heyde B. Development and evaluation of a novel vegfr2-targeted nanoscale ultrasound contrast agents. 2016; 9790:97900. 10.1117/12.2214812.

[58] An J, Zhang J, Dong F, Yin J, Feng F, Guo W, Huang S, Wang D, Dang J, Zhang J (2022). Arterial labeling ultrasound subtraction angiography (alusa) based on acoustic phase-change nanodroplets. Small.

[59] Manohar S, Gambhir SS (2020). Clinical photoacoustic imaging. Photoacoustics.

[60] Lin L, Wang LV (2021). Photoacoustic imaging. Adv Exp Med Biol.

[61] He C, Zhu J, Zhang H, Qiao R, Zhang R (2022). Photoacoustic imaging probes for theranostic applications. Biosensors (Basel)..

[62] Seong M, Chen SL (2020). Recent advances toward clinical applications of photoacoustic microscopy: a review. Sci China Life Sci.

[63] Zhang C, Liu G, Xie Z, Shu Z, Ren Z, Yao Q (2021). Vascular recognition system based on photoacoustic detection. J Laser Appl.

[64] Park B, Oh D, Kim J, Kim C (2023). Functional photoacoustic imaging: from nano- and micro- to macro-scale. Nano Converg.

[65] Yao DK, Maslov K, Shung KK, Zhou Q, Wang LV (2010). In vivo label-free photoacoustic microscopy of cell nuclei by excitation of DNA and RNA. Opt Lett.

[66] Lee DY, Kim JY, Lee Y, Lee S, Miao W, Kim HS, Min JJ, Jon S (2017). Black pigment gallstone inspired platinum-chelated bilirubin nanoparticles for combined photoacoustic imaging and photothermal therapy of cancers. Angew Chem Int Ed Engl.

[67] Yan Y, Basij M, Garg A, Varrey A, Alhousseini A, Hsu R, Hernandez-Andrade E, Romero R, Hassan SS, Mehrmohammadi M (2021). Spectroscopic photoacoustic imaging of cervical tissue composition in excised human samples. PLoS ONE.

[68] Maccuaig WM, Jones MA, Abeyakoon O, Mcnally LR (2020). Development of multispectral optoacoustic tomography as a clinically translatable modality for cancer imaging. Radiol Imaging Cancer.

[69] Zeng L, Ma G, Lin J, Huang P (2018). Photoacoustic probes for molecular detection: recent advances and perspectives. Small.

[70] Li W, Chen X (2015). Gold nanoparticles for photoacoustic imaging. Nanomedicine (Lond).

[71] Du J, Yang S, Qiao Y, Lu H, Dong H (2021). Recent progress in near-infrared photoacoustic imaging. Biosens Bioelectron.

[72] Pu K, Shuhendler AJ, Jokerst JV, Mei J, Gambhir SS, Bao Z, Rao J (2014). Semiconducting polymer nanoparticles as photoacoustic molecular imaging probes in living mice. Nat Nanotechnol.

[73] Zeng Y, Dou T, Ma L, Ma J (2022). Biomedical photoacoustic imaging for molecular detection and disease diagnosis: "always-on" and "turn-on" probes. Adv Sci (Weinh).

[74] Liu C, Wang D, Zhan Y, Yan L, Lu Q, Chang MYZ, Luo J, Wang L, Du D, Lin Y (2018). Switchable photoacoustic imaging of glutathione using MnO_2_ nanotubes for cancer diagnosis. Acs Appl Mater Interfaces.

[75] Gong F, Cheng L, Yang N, Jin Q, Tian L, Wang M, Li Y, Liu Z (2018). Bimetallic oxide MnMOo(x) nanorods for in vivo photoacoustic imaging of GSH and tumor-specific photothermal therapy. Nano Lett.

[76] Knox HJ, Hedhli J, Kim TW, Khalili K, Dobrucki LW, Chan J (2017). A bioreducible *n*-oxide-based probe for photoacoustic imaging of hypoxia. Nat Commun.

[77] Jarrett DY, Ali M, Chaudry G (2013). Imaging of vascular anomalies. Dermatol Clin.

[78] Hyodoh H, Hori M, Akiba H, Tamakawa M, Hyodoh K, Hareyama M (2005). Peripheral vascular malformations: imaging, treatment approaches, and therapeutic issues. Radiographics.

[79] Hayakawa N, Kodera S, Ohki N, Kanda J (2019). Efficacy and safety of endovascular therapy by diluted contrast digital subtraction angiography in patients with chronic kidney disease. Heart Vessels.

[80] Ma Y, Wan J, Qian K, Geng S, He N, Zhou G, Zhao Y, Yang X (2014). The studies on highly concentrated complex dispersions of gold nanoparticles and temperature-sensitive nanogels and their application as new blood-vessel-embolic materials with high-resolution angiography. J Mater Chem B.

[81] Pramod KE, Um W, Park JH (2020). Recent developments in pathological ph-responsive polymeric nanobiosensors for cancer theranostics. Front Bioeng Biotechnol.

[82] Sharifi M, Avadi MR, Attar F, Dashtestani F, Ghorchian H, Rezayat SM, Saboury AA, Falahati M (2019). Cancer diagnosis using nanomaterials based electrochemical nanobiosensors. Biosens Bioelectron.

[83] Sargazi S, Fatima I, Hassan KM, Mohammadzadeh V, Arshad R, Bilal M, Rahdar A, Diez-Pascual AM, Behzadmehr R (2022). Fluorescent-based nanosensors for selective detection of a wide range of biological macromolecules: a comprehensive review. Int J Biol Macromol.

[84] Li W, Ma C, Song Y, Hong C, Qiao X, Yin B (2020). Sensitive detection of carcinoembryonic antigen (CEA) by a sandwich-type electrochemical immunosensor using mof-ce@ha/ag-hrp-ab(2) as a nanoprobe. Nanotechnology.

[85] Hage AN, Chick J, Srinivasa RN, Bundy JJ, Chauhan NR, Acord M, Gemmete JJ (2018). Treatment of venous malformations: the data, where we are, and how it is done. Tech Vasc Interv Radiol.

[86] Zaki GM, Kan P, Britz GW (2019). Curative embolization of arteriovenous malformations. World Neurosurg.

[87] Hu J, Albadawi H, Chong BW, Deipolyi AR, Sheth RA, Khademhosseini A, Oklu R (2019). Advances in biomaterials and technologies for vascular embolization. Adv Mater.

[88] Dong H, Yang D, Hu Y, Song X (2022). Recent advances in smart nanoplatforms for tumor non-interventional embolization therapy. J Nanobiotechnol.

[89] Vollherbst DF, Chapot R, Bendszus M, Mohlenbruch MA (2022). Glue, onyx, squid or phil? Liquid embolic agents for the embolization of cerebral arteriovenous malformations and dural arteriovenous fistulas. Clin Neuroradiol.

[90] El-Abtah ME, Petitt JC, Kashkoush A, Achey R, Bain MD, Moore NZ (2022). Endovascular management of arteriovenous malformation-associated intracranial aneurysms: a systematic literature review. World Neurosurg.

[91] Lazzaro MA, Badruddin A, Zaidat OO, Darkhabani Z, Pandya DJ, Lynch JR (2011). Endovascular embolization of head and neck tumors. Front Neurol.

[92] Brill RM, Guntau M, Wildgruber M, Brill E, Stangl F, Taute BM, Ukkat J, Goldann C, Wohlgemuth WA (2021). Safety and effectiveness of ethylene vinyl alcohol copolymer embolization of peripheral high-flow arteriovenous malformations: results of a prospective study. J Vasc Interv Radiol.

[93] Sampei K, Hashimoto N, Kazekawa K, Tsukahara T, Iwata H, Takaichi S (1996). Histological changes in brain tissue and vasculature after intracarotid infusion of organic solvents in rats. Neuroradiology.

[94] Yamashita K, Taki W, Iwata H, Nakahara I, Nishi S, Sadato A, Matsumoto K, Kikuchi H (1994). Characteristics of ethylene vinyl alcohol copolymer (eval) mixtures. Ajnr Am J Neuroradiol.

[95] Grasso RF, Cazzato RL, Luppi G, Faiella E, Del VR, Giurazza F, Borzomati D, Coppola R, Zobel BB (2012). Pancreatic arteriovenous malformation involving the duodenum embolized with ethylene-vinyl alcohol copolymer (onyx). Cardiovasc Intervent Radiol.

[96] Albanese G, Kondo KL (2010). Pharmacology of sclerotherapy. Semin Intervent Radiol.

[97] Lee CH, Chen SG (2005). Direct percutaneous ethanol instillation for treatment of venous malformation in the face and neck. Br J Plast Surg.

[98] Mason KP, Michna E, Zurakowski D, Koka BV, Burrows PE (2000). Serum ethanol levels in children and adults after ethanol embolization or sclerotherapy for vascular anomalies. Radiology.

[99] Ko G, Choi JW, Lee N, Kim D, Hyeon T, Kim H (2022). Recent progress in liquid embolic agents. Biomaterials.

[100] Lord J, Britton H, Spain SG, Lewis AL (2020). Advancements in the development on new liquid embolic agents for use in therapeutic embolisation. J Mater Chem B.

[101] Zhao Y, Zheng C, Wang Q, Fang J, Zhou G, Zhao H, Yang Y, Xu H, Feng G, Yang X (2011). Permanent and peripheral embolization: temperature-sensitive p(*n*-isopropylacrylamide-*co*-butyl methylacrylate) nanogel as a novel blood-vessel-embolic material in the interventional therapy of liver tumors. Adv Funct Mater.

[102] Alexander A, Ajazuddin, Khan J, Saraf S, Saraf S. Polyethylene glycol (peg)-poly(*n*-isopropylacrylamide) (pnipaam) based thermosensitive injectable hydrogels for biomedical applications. Eur J Pharm Biopharm. 2014;88:575–585. 10.1016/j.ejpb.2014.07.005. 10.1016/j.ejpb.2014.07.005 25092423

[103] Dai F, Tang L, Yang J, Zhao X, Liu W, Chen G, Xiao F, Feng X (2009). Fast thermoresponsive bab-type hema/nipaam triblock copolymer solutions for embolization of abnormal blood vessels. J Mater Sci Mater Med.

[104] Zhao Y, Zheng C, Wang Q, Fang J, Zhou G, Zhao H, Yang Y, Xu H, Feng G, Yang X (2011). Permanent and peripheral embolization: temperature-sensitive p(*n*-isopropylacrylamide-*co*-butyl methylacrylate) nanogel as a novel blood-vessel-embolic material in the interventional therapy of liver tumors. Adv Funct Mater.

[105] Zhao H, Zheng C, Feng G, Zhao Y, Liang H, Wu H, Zhou G, Liang B, Wang Y, Xia X (2013). Temperature-sensitive poly (*n*-isopropylacrylamide-*co*-butyl methylacrylate) nanogel as an embolic agent: distribution, durability of vascular occlusion, and inflammatory reactions in the renal artery of rabbits. AJNR Am J Neuroradiol.

[106] Ma Y, Wan J, Qian K, Geng S, He N, Zhou G, Zhao Y, Yang X (2014). The studies on highly concentrated complex dispersions of gold nanoparticles and temperature-sensitive nanogels and their application as new blood-vessel-embolic materials with high-resolution angiography. J Mater Chem B.

[107] Bracaglia LG, Fisher JP (2015). Extracellular matrix-based biohybrid materials for engineering compliant, matrix-dense tissues. Adv Healthc Mater.

[108] Frantz C, Stewart KM, Weaver VM (2010). The extracellular matrix at a glance. J Cell Sci.

[109] Traverse JH, Henry TD, Dib N, Patel AN, Pepine C, Schaer GL, Dequach JA, Kinsey AM, Chamberlin P, Christman KL (2019). First-in-man study of a cardiac extracellular matrix hydrogel in early and late myocardial infarction patients. JACC Basic Transl Sci.

[110] Saldin LT, Cramer MC, Velankar SS, White LJ, Badylak SF (2017). Extracellular matrix hydrogels from decellularized tissues: structure and function. Acta Biomater.

[111] Hu J, Altun I, Zhang Z, Albadawi H, Salomao MA, Mayer JL, Hemachandra L, Rehman S, Oklu R (2020). Bioactive-tissue-derived nanocomposite hydrogel for permanent arterial embolization and enhanced vascular healing. Adv Mater.

[112] Van Damme A, Seront E, Dekeuleneer V, Boon LM, Vikkula M (2020). New and emerging targeted therapies for vascular malformations. Am J Clin Dermatol.

[113] Karar J, Maity A (2011). Pi3k/akt/mtor pathway in angiogenesis. Front Mol Neurosci.

[114] Ahmad A, Nawaz MI (2022). Molecular mechanism of vegf and its role in pathological angiogenesis. J Cell Biochem.

[115] Huang WQ, Zou Y, Tian Y, Ma XF, Zhou QY, Li ZY, Gong SX, Wang AP (2022). Mammalian target of rapamycin as the therapeutic target of vascular proliferative diseases: past, present, and future. J Cardiovasc Pharmacol.

[116] Lee JC, Modiri O, England RW, Shawber CJ, Wu JK (2021). Propranolol therapy in infantile hemangioma: it is not just about the beta. Plast Reconstr Surg.

[117] Samad A, Ullah Z, Alam MI, Wais M, Shams MS (2009). Transdermal drug delivery system: patent reviews. Recent Pat Drug Deliv Formul.

[118] Marwah H, Garg T, Goyal AK, Rath G (2016). Permeation enhancer strategies in transdermal drug delivery. Drug Deliv.

[119] Rehman K, Zulfakar MH (2013). Recent advances in gel technologies for topical and transdermal drug delivery. Drug Dev Ind Pharm.

[120] Leducq S, Caille A, Barbarot S, Bénéton N, Bessis D, Boccara O, Bursztejn A, Chiaverini C, Dompmartin A, Droitcourt C (2019). Topical sirolimus 0.1% for treating cutaneous microcystic lymphatic malformations in children and adults (topical): protocol for a multicenter phase 2, within-person, randomized, double-blind, vehicle-controlled clinical trial. Trials..

[121] Nagata E, Kashiwagura Y, Okada E, Tanaka S, Sano S, Nishida M, Hayano S, Iwashima S, Hakamata A, Odagiri K (2022). Efficacy and safety of propranolol cream in infantile hemangioma: a prospective pilot study. J Pharmacol Sci.

[122] Badia P, Ricci K, Gurria JP, Dasgupta R, Patel M, Hammill A (2020). Topical sirolimus for the treatment of cutaneous manifestations of vascular anomalies: a case series. Pediatr Blood Cancer.

[123] Prausnitz MR, Langer R (2008). Transdermal drug delivery. Nat Biotechnol.

[124] Guan Y, Zuo T, Chang M, Zhang F, Wei T, Shao W, Lin G (2015). Propranolol hydrochloride-loaded liposomal gel for transdermal delivery: characterization and in vivo evaluation. Int J Pharm.

[125] Chen ZG, Zheng JW, Yuan ML, Zhang L, Yuan WE (2015). A novel topical nano-propranolol for treatment of infantile hemangiomas. Nanomed Nanotechnol Biol Med.

[126] Padula C, Nicoli S, Pescina S, Santi P (2019). Thin polymeric films for the topical delivery of propranolol. Colloids Surf B.

[127] Zeng L, Tao C, Liu Z, Zhang J, Zhang M, Zhang J, Fang S, Ma X, Song H, Zhou X (2020). Preparation and evaluation of cubic nanoparticles for improved transdermal delivery of propranolol hydrochloride. AAPS Pharmscitech..

[128] Zhang X, Wang Y, Chi J, Zhao Y (2020). Smart microneedles for therapy and diagnosis. Research (Wash D C).

[129] Waghule T, Singhvi G, Dubey SK, Pandey MM, Gupta G, Singh M, Dua K (2019). Microneedles: a smart approach and increasing potential for transdermal drug delivery system. Biomed Pharmacother.

[130] Yang D, Chen M, Sun Y, Jin Y, Lu C, Pan X, Quan G, Wu C (2021). Microneedle-mediated transdermal drug delivery for treating diverse skin diseases. Acta Biomater.

[131] Sartawi Z, Blackshields C, Faisal W (2022). Dissolving microneedles: applications and growing therapeutic potential. J Control Release.

[132] He J, Zhang Z, Zheng X, Li L, Qi J, Wu W, Lu Y (2021). Design and evaluation of dissolving microneedles for enhanced dermal delivery of propranolol hydrochloride. Pharmaceutics.

[133] Mao J, Wang H, Xie Y, Fu Y, Li Y, Liu P, Du H, Zhu J, Dong L, Hussain M (2020). Transdermal delivery of rapamycin with poor water-solubility by dissolving polymeric microneedles for anti-angiogenesis. J Mater Chem B.

[134] Fu Y, Yang ZG, Zhao LY (2017). Angiogenesis characteristics of infantile hemangioma and feasibility observation of transplantation model of human hemangioma on mice. Eur Rev Med Pharmacol Sci.

[135] Mccoll BK, Stacker SA, Achen MG (2004). Molecular regulation of the vegf family—inducers of angiogenesis and lymphangiogenesis. APMIS.

[136] Saikia Q, Reeve H, Alzahrani A, Critchley WR, Zeqiraj E, Divan A, Harrison MA, Ponnambalam S (2023). Vegfr endocytosis: implications for angiogenesis. Prog Mol Biol Transl Sci.

[137] Cullion K, Petishnok LC, Koo H, Harty B, Melero-Martin JM, Kohane DS (2021). Targeting nanoparticles to bioengineered human vascular networks. Nano Lett.

[138] Kalluri R, Lebleu VS (2020). The biology, function, and biomedical applications of exosomes. Science..

[139] Li H, Wang X, Guo X, Wan Q, Teng Y, Liu J (2022). Development of rapamycin-encapsulated exosome-mimetic nanoparticles-in-plga microspheres for treatment of hemangiomas. Biomed Pharmacother.

[140] Yu Y, Duan J, Yu Y, Li Y, Liu X, Zhou X, Ho KF, Tian L, Sun Z (2014). Silica nanoparticles induce autophagy and autophagic cell death in hepg2 cells triggered by reactive oxygen species. J Hazard Mater.

[141] Duan J, Yu Y, Yu Y, Li Y, Wang J, Geng W, Jiang L, Li Q, Zhou X, Sun Z (2014). Silica nanoparticles induce autophagy and endothelial dysfunction via the pi3k/akt/mtor signaling pathway. Int J Nanomedicine.

[142] Rangel M, Kong J, Bhatt V, Khayati K, Guo JY (2022). Autophagy and tumorigenesis. FEBS J.

[143] Wu H, Wang X, Liang H, Zheng J, Huang S, Zhang D (2020). Enhanced efficacy of propranolol therapy for infantile hemangiomas based on a mesoporous silica nanoplatform through mediating autophagy dysfunction. Acta Biomater.

[144] Brauer JA, Geronemus RG (2013). Laser treatment in the management of infantile hemangiomas and capillary vascular malformations. Tech Vasc Interv Radiol.

[145] Overchuk M, Weersink RA, Wilson BC, Zheng G (2023). Photodynamic and photothermal therapies: synergy opportunities for nanomedicine. ACS Nano.

[146] Wang Y, Meng HM, Li Z (2021). Near-infrared inorganic nanomaterial-based nanosystems for photothermal therapy. Nanoscale.

[147] Cullion K, Ostertag-Hill CA, Pan M, Timko B, Boscolo E, Kohane DS (2023). Ablation of venous malformations by photothermal therapy with intravenous gold nanoshells. Nano Lett.

[148] Jiang Y, Liu J, Qin J, Lei J, Zhang X, Xu Z, Li W, Liu X, Wang R, Li B (2022). Light-activated gold nanorods for effective therapy of venous malformation. Mater Today Bio.

[149] Li X, Lovell JF, Yoon J, Chen X (2020). Clinical development and potential of photothermal and photodynamic therapies for cancer. Nat Rev Clin Oncol.

[150] Lou X, Zhang G, Song N, Yang Y (2022). Supramolecular materials based on aiegens for photo-assisted therapy. Biomaterials.

[151] Kwiatkowski S, Knap B, Przystupski D, Saczko J, Kedzierska E, Knap-Czop K, Kotlinska J, Michel O, Kotowski K, Kulbacka J (2018). Photodynamic therapy—mechanisms, photosensitizers and combinations. Biomed Pharmacother.

[152] Orbay H, Li Y, Xiao W, Cherry SR, Lam K, Sahar DE (2016). Developing a nanoparticle-delivered high-efficacy treatment for infantile hemangiomas using a mouse hemangioendothelioma model. Plast Reconstr Surg.

[153] Guan Y, Zuo T, Chang M, Zhang F, Wei T, Shao W, Lin G (2015). Propranolol hydrochloride-loaded liposomal gel for transdermal delivery: characterization and in vivo evaluation. Int J Pharm.

[154] Li H, Teng Y, Xu X, Liu J (2018). Enhanced rapamycin delivery to hemangiomas by lipid polymer nanoparticles coupled with anti-vegfr antibody. Int J Mol Med.

[155] Li H, Teng Y, Sun J, Liu J (2017). Inhibition of hemangioma growth using polymer–lipid hybrid nanoparticles for delivery of rapamycin. Biomed Pharmacother.

[156] Guo X, Zhu X, Liu D, Gong Y, Sun J, Dong C (2017). Continuous delivery of propranolol from liposomes-in-microspheres significantly inhibits infantile hemangioma growth. Int J Nanomed.

[157] Cullion K, Petishnok LC, Koo H, Harty B, Melero-Martin JM, Kohane DS (2021). Targeting nanoparticles to bioengineered human vascular networks. Nano Lett.

[158] Li X, Ren X, Liang J, Ma W, Wang Z, Yang Z (2017). Delivery of sodium morrhuate to hemangioma endothelial cells using immunoliposomes conjugated with anti-vegfr2/kdr antibody. Int J Nanomed.

[159] Liu L, Li X, Zhao Q, Yang L, Jiang X (2022). Pathogenesis of port-wine stains: directions for future therapies. Int J Mol Sci..

[160] Mäkinen T, Boon LM, Vikkula M, Alitalo K (2021). Lymphatic malformations: genetics, mechanisms and therapeutic strategies. Circ Res.

